# Genome-Wide Meta-Analysis of Five Asian Cohorts Identifies *PDGFRA* as a Susceptibility Locus for Corneal Astigmatism

**DOI:** 10.1371/journal.pgen.1002402

**Published:** 2011-12-01

**Authors:** Qiao Fan, Xin Zhou, Chiea-Chuen Khor, Ching-Yu Cheng, Liang-Kee Goh, Xueling Sim, Wan-Ting Tay, Yi-Ju Li, Rick Twee-Hee Ong, Chen Suo, Belinda Cornes, Mohammad Kamran Ikram, Kee-Seng Chia, Mark Seielstad, Jianjun Liu, Eranga Vithana, Terri L. Young, E.-Shyong Tai, Tien-Yin Wong, Tin Aung, Yik-Ying Teo, Seang-Mei Saw

**Affiliations:** 1 School of Public Health, National University of Singapore, Singapore, Singapore; 2 Genome Institute of Singapore, Agency for Science, Technology, and Research, Singapore, Singapore; 3 Singapore Eye Research Institute, Singapore National Eye Centre, Singapore, Singapore; 4 Centre for Molecular Epidemiology, National University of Singapore, Singapore, Singapore; 5 Department of Pediatrics, National University of Singapore, Singapore, Singapore; 6 Department of Ophthalmology, National University of Singapore, Singapore, Singapore; 7 Duke–National University of Singapore Graduate Medical School, Singapore, Singapore; 8 Department of Medical Oncology, National Cancer Centre Singapore, Singapore, Singapore; 9 Center for Human Genetics, Duke University Medical Center, Durham, North Carolina, United States of America; 10 Department of Biostatistics and Bioinformatics, Duke University Medical Center, Durham, North Carolina, United States of America; 11 NUS Graduate School for Integrative Science and Engineering, National University of Singapore, Singapore, Singapore; 12 Department of Ophthalmology, Erasmus Medical Center, Rotterdam, The Netherlands; 13 Institute for Human Genetics and Department of Laboratory Medicine, University of California San Francisco, San Francisco, California, United States of America; 14 Department of Medicine, National University of Singapore, Singapore, Singapore; 15 Centre for Eye Research Australia, University of Melbourne, Melbourne, Australia; 16 Department of Statistics and Applied Probability, National University of Singapore, Singapore, Singapore; Harvard University, United States of America

## Abstract

Corneal astigmatism refers to refractive abnormalities and irregularities in the curvature of the cornea, and this interferes with light being accurately focused at a single point in the eye. This ametropic condition is highly prevalent, influences visual acuity, and is a highly heritable trait. There is currently a paucity of research in the genetic etiology of corneal astigmatism. Here we report the results from five genome-wide association studies of corneal astigmatism across three Asian populations, with an initial discovery set of 4,254 Chinese and Malay individuals consisting of 2,249 cases and 2,005 controls. Replication was obtained from three surveys comprising of 2,139 Indians, an additional 929 Chinese children, and an independent 397 Chinese family trios. Variants in *PDGFRA* on chromosome 4q12 (lead SNP: rs7677751, allelic odds ratio = 1.26 (95% CI: 1.16–1.36), *P*_meta_ = 7.87×10^−9^) were identified to be significantly associated with corneal astigmatism, exhibiting consistent effect sizes across all five cohorts. This highlights the potential role of variants in *PDGFRA* in the genetic etiology of corneal astigmatism across diverse Asian populations.

## Author Summary

Corneal astigmatism is associated with reduced visual acuity and an increased risk of developing refractive amblyopia. Although it is highly heritable, there is no prior study on the genetic etiology of corneal astigmatism. Our genome-wide meta-analysis across 8,513 individuals in five genome-wide surveys from three genetically diverse populations in Asia reveals that genetic variants in the *PDGFRA* gene on chromosome 4q12 is significantly associated with corneal astigmatism. These polymorphisms in the *PDGFRA* gene exhibit strong and consistent effects over all five Asian cohorts. *PDGFRA* is a receptor for platelet-derived growth factor, which is expressed in many retinal tissues in the eyes and appears to contribute to ocular development. Results from our study further suggest the potential role of *PDGFRA* in the regulation of corneal biometrics.

## Introduction

Astigmatism is a condition where light rays are prevented from focusing at a single point in the eye, resulting in blurred vision at any near or far distance. While astigmatism comprises cornea and non-corneal components, it typically results from the unequal curvature of two principle meridians in the anterior surface of the cornea known as corneal astigmatism [1], [2]. The presence of a high degree of astigmatism during early development is believed to be associated with refractive amblyopia [3], [4], [5], as evidenced by decreased best-corrected visual acuity which cannot be remedied by external corrective lenses. Early abnormal visual input caused by uncorrected astigmatism can lead to orientation-dependent visual deficits, despite optical correction of visual acuity later in life [6]. In addition, it has been suggested that optical blurring by astigmatism may predispose the development of myopia, commonly known as nearsightedness [7], [8], [9], [10].

Astigmatism is highly prevalent across most populations and poses a significant burden to global public health with at least 1 in 3 adults above 30 years of age suffering from astigmatism of −0.5 diopters (D) or less [11]. The reported age-adjusted prevalence of astigmatism was 37.8% for Chinese adults [12], 54.8% in rural Asian Indians [13], 37% (≤−0.75D) for Caucasian in Australia [14] and 36.2% in the US [15]. The prevalence of astigmatism in children varies considerably across different studies and ethnic groups. For instance, the prevalence of astigmatism (≤−0.75D) in school-children ranges from 13.6% in Australia [16], 20% in Northern Ireland [17], 28.4% for Singapore school children [8], to 42.7% for Chinese children in urban China [18].

Although the precise cause of astigmatism is unknown, genetic factors have been implicated in the etiology of corneal astigmatism. Studies have reported a higher risk of developing astigmatism in individuals whose sibling or parents have astigmatism [11]. Evidence from twin studies suggests a genetic etiology in astigmatism development, with the estimated heritability ranging from 30% to 60% [19], [20], [21], [22], [23]. For instance, Hammond and colleagues [21] investigated the inheritance of astigmatism for 226 monzygotic (MZ) and 280 dizygotic (DZ) twins in the United Kingdom and found genetic effects accounted for 42% to 61% of the variation in corneal astigmatism. While most of the twin studies have been conducted in Caucasian populations, a study on Chinese twins in Taiwan reported a heritability estimate of 46% for corneal astigmatism, suggesting that genetic factors account for a similar extent in the etiology of astigmatism for Asian populations [22]. However, no genetic loci have been systematically and consistently identified to be implicated in the development of corneal astigmatism.

Here we report the findings from the meta-analyses of five genome-wide association studies (GWAS) performed in 8,513 individuals from three Asian populations. The discovery phase of our study comprises 4,254 individuals from two population-based GWAS performed in adults of Chinese and Malay ethnicities from the Singapore Prospective Study Program (SP2) and the Singapore Malay Eye Study (SiMES) respectively. The replication phase comprises of data from three other genome-wide surveys of: (i) 2,139 Indian adults from the Singapore Indian Eye Study (SINDI); (ii) 929 Chinese school children from the Singapore Cohort Study of the Risk Factors for Myopia (SCORM); and (iii) 397 Chinese trios of parents and astigmatic offsprings from the Singaporean Chinese in the Strabismus, Amblyopia and Refractive Error Study (STARS).

## Results

The characteristics of the post-QC samples from the five studies are summarized in Table 1. The post-QC SP2 dataset comprised 2,016 adults, of which 1,231 individuals had corneal astigmatism (≤−0.75 D) and 785 subjects were defined as non-astigmatic controls. The post-QC SiMES dataset comprised 2,238 adults (1,018 cases and 1,220 controls). In total, 462,518 and 515,712 autosomal genotyped SNPs passed stringent quality control criteria for SP2 and SiMES respectively and the genome-wide meta-analysis was conducted on 460,528 SNPs present in both studies.

**Table 1:** Characteristics of the participants in five studies.

Cohorts	SP2	SIMES	SINDI	SCORM	STARS
**Individuals genotyped**	2687	3280	2953	1116	1451 (440 families)
**Individuals after QC**	2434	2542	2538	1008	1351 (407 families)
**Individuals in GWAS**	2016	2238	2139	929	1191 (397 parents-trios)
**Male (%)**	45.8%	48.9%	51.2%	51.8%	52.3%^a^
**Age (SD)**	47.9 (11.2)	57.7 (10.7)	55.9 (8.9)	10.8 (0.8)	7.5 (3.8)^a^
**Cases**	1231	1018	825	760	NA
**Controls**	785	1220	1314	169	NA
**Corneal cylinder power** b **(SD)**					
**Cases**	−1.38 (0.73)	−1.37 (0.94)	−1.21 (0.58)	−1.52 (0.68)	1.30 (0.78)*
**Controls**	−0.48 (0.15)	−0.46 (0.15)	−0.46 (0.16)	−0.47 (0.14)	NA

a Information of gender and age is based on data from offspring with corneal astigmatism in STARS family cohort.

b Averaged across both eyes; NA, not available; Age is in years.

SP2 - Singapore Prospective Study Program; SiMES - Singapore Malay Eye Study; SINDI - Singapore Indian Eye Study; SCORM - Singapore Cohort study of the Risk factors for Myopia; STARS - Singaporean Chinese in the Strabismus, Amblyopia and Refractive Error Study.

There was no evidence of over-inflation of statistical significances due to population structure in either of the discovery cohorts (SP2 λ_GC_ = 1.006, SiMES λ_GC_ = 1.007) or in the meta-analysis of both studies (overall λ_GC_ = 1.007). Suggestive evidence of association (defined as 10^−6^<*P*-value<10^−5^) were seen in each of SP2 and SiMES (Figure S1A and S1B), as well as in the meta-analysis of SP2 and SiMES where a collection of SNPs deviated from their expected distributions in the quantile-quantile plots of the *P*-values (Figure S1C).

None of the SNPs in the discovery meta-analysis attained genome-wide significance of *P*-value<5×10^−8^. Seven SNPs exhibited evidence stronger than *P*-value<1.0×10^−5^ and these were found to cluster in the platelet-derived growth factor receptor alpha (*PDGFRA*) gene on chromosome 4q12 (lowest *P* = 9.44×10^−7^ at rs7677751; Table 2; Figure S2). Interestingly, these SNPs are located within the MYP9 region identified previously as a candidate locus for myopia through linkage scans [24].

**Table 2:** Top association signals in the discovery and replication GWAS of corneal astigmatism.

					Discovery cohorts					Replication cohorts								
					SP2 (n = 2,016)Case (1,231)Control (785)		SiMES (n = 2,238)Case (1,018)Control(1,220)		Meta-analysis	SINDI(n = 2,139)Case (825)Control (1,314)		SOCRM (n = 929)Case (760)Control(169)		STARS (n = 1,191)(397 trios)		Overall meta-analysis		
SNP	GENE	BP^a^	EA^b^	EAF^c^	OR(s.e.^d^)	*P*	OR(s.e.)	*P*	*P*	OR(s.e.)	*P*	OR(s.e.)	*P*	OR(s.e.)	*P*	OR(s.e.)	*P*	*P_het_* e
rs17084051	54782338	A		0.23	1.35(0.08)	3.68E-04	1.26(0.07)	1.08E-03	1.63E-06	1.14(0.07)	7.77E-02	1.23(0.15)	1.68E-01	1.04(0.12)	7.55E-01	1.21(0.04)	2.16E-06	2.46E-01
rs7677751	PDGFRA	54819217	T	0.22	1.35(0.09)	3.76E-04	1.27(0.07)	6.26E-04	9.44E-07	1.23(0.07)	4.09E-03	1.22(0.15)	2.00E-01	1.12(0.13)	3.74E-01	1.26(0.04)	7.87E-09	7.86E-01
rs2307049	PDGFRA	54824911	A	0.23	1.31(0.09)	2.17E-03	1.26(0.07)	7.55E-04	5.55E-06	1.27(0.07)	1.08E-03	1.14(0.15)	4.03E-01	1.14(0.13)	3.17E-01	1.25(0.04)	1.58E-08	8.85E-01
rs7660560	PDGFRA	54829151	A	0.23	1.32(0.09)	1.46E-03	1.28(0.07)	5.34E-04	2.65E-06	1.26(0.07)	1.37E-03	1.13(0.15)	4.22E-01	1.14(0.13)	3.20E-01	1.27(0.04)	1.15E-08	8.39E-01
rs2228230	PDGFRA	54846797	T	0.16	1.41(0.10)	5.94E-04	1.26(0.08)	3.19E-03	8.56E-06	1.19(0.07)	1.67E-02	1.05(0.17)	7.69E-01	1.06(0.14)	6.71E-01	1.24(0.04)	1.43E-06	3.62E-01
rs4864872	PDGFRA	54847041	T	0.16	1.41(0.10)	5.94E-04	1.26(0.08)	3.19E-03	8.56E-06	1.19(0.07)	1.49E-02	1.05(0.17)	7.69E-01	1.06(0.14)	6.71E-01	1.24(0.04)	1.24E-06	3.70E-01
rs3690	PDGFRA	54856570	C	0.16	1.40(0.10)	6.25E-04	1.29(0.08)	1.53E-03	3.93E-06	1.18(0.07)	2.17E-02	1.03(0.17)	8.58E-01	1.09(0.14)	5.28E-01	1.25(0.04)	7.77E-07	3.91E-01

a Base pair positions are indicated according to the NCBI build 136 (hg18);

b Effect allele;

c Average effect allele frequency in the discovery cohort;

d Standard error for odds ratios;

e *P*-value for heterogeneity I^2^ between five study cohorts.

SP2 - Singapore Prospective Study Program; SiMES - Singapore Malay Eye Study; SINDI - Singapore Indian Eye Study; SCORM - Singapore Cohort study of the Risk factors for Myopia; STARS - Singaporean Chinese in the Strabismus, Amblyopia and Refractive Error Study.

**Figure 1:**
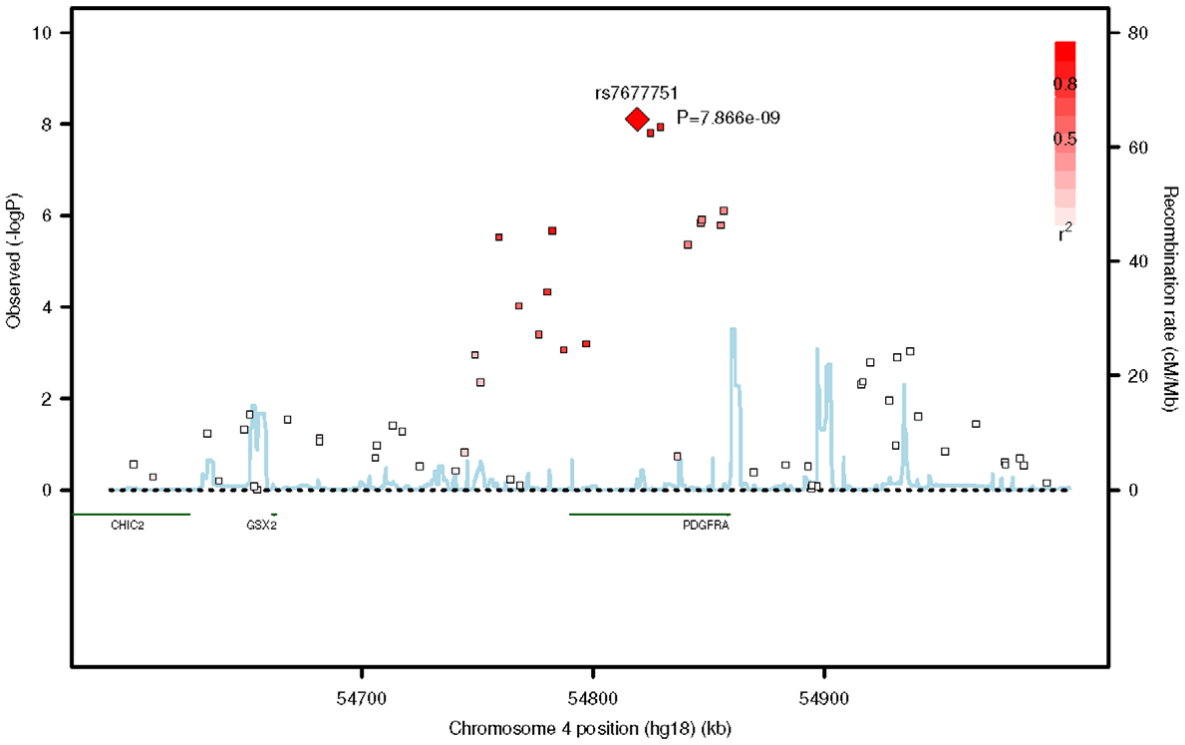
Regional plot of the association signals from the meta-analysis of the five GWAS cohorts around the *PDGFRA* gene locus. A region of 400 kb around the lead SNP (rs7677751, red diamond) is shown. The LD between the lead SNP and the neighbouring SNPs is represented by the shading of the squares, with increasing shade of red indicating higher LD as measured by r^2^. The blue lines represent the recombination rates of JPT+CHB panels from HapMap II.

**Figure 2:**
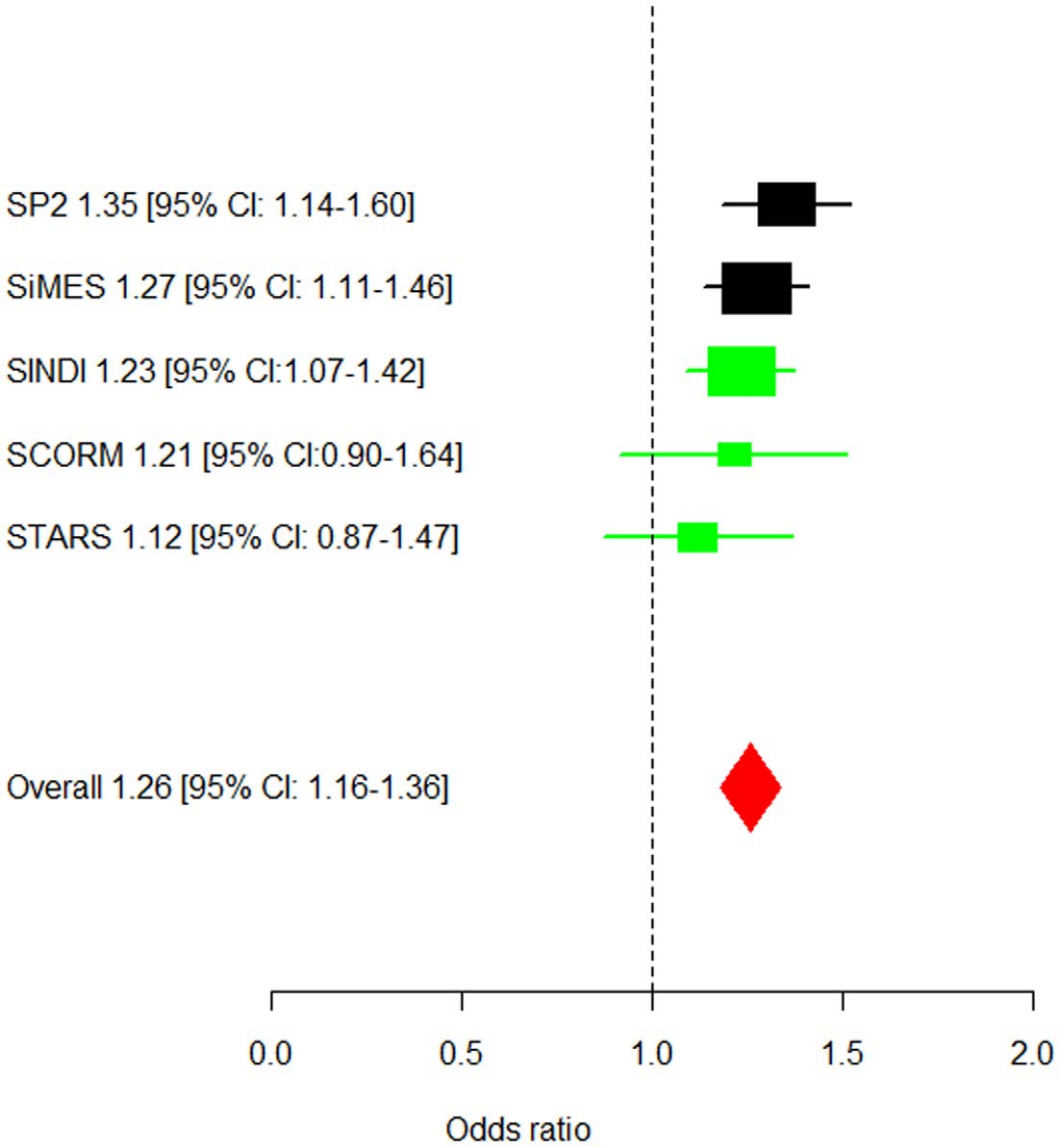
Forest plot of the estimated allelic odds ratios for the lead SNP rs7677751. The allelic odd ratios for allele T of rs7677751 and 95% confidence intervals are presented for the five studies separately (black rectangles for discovery studies, green rectangles for replication studies), the meta-analyses during the discovery (black diamond) and replication (green diamond) phases, and for the overall meta-analysis across all five studies (red diamond).

In the replication phase with the three additional GWAS cohorts, three SNPs in *PDGFRA* (rs7677751, rs2307049 and rs7660560) attained genome-wide significance in the combined analysis (Table 2) with the lead SNP rs7677751 from the discovery phase remaining as the strongest signal in the combined analysis (*P* = 7.87×10^−9^; Figure 1). All seven SNPs from the discovery phase exhibited *P*-values<0.05 in SINDI but not in SCORM or STARS. However the direction and magnitude of the effect sizes at these seven SNPs in all three replication cohorts were highly similar to those seen in the discovery populations of SP2 and SiMES (Table 2, Figure 2). No significant evidence of effect size heterogeneity was detected across the SNPs (heterogeneity I^2^ *P*-value≥0.246), and the minor allele frequencies of these SNPs are consistently similar across all five studies (Table S1). A genome-wide meta-analysis of the combined five cohorts did not yield any additional locus with genome-wide significance (see Figure S3 for QQ and Manhattan plots, λ_GC_ = 1.002; Table S2).

At the lead SNP rs7677751 in *PDGFRA*, the frequency of the risk T-allele ranged from 0.19 to 0.26 in the five cohorts and conferred a 26% higher risk of corneal astigmatism than the C allele (OR = 1.26, 95% CI = 1.16–1.36) in the meta-analysis across all five studies. This SNP alone explains 0.41% of the variation in corneal cylinder power. In addition, a general genetic model identified that the 5.5% of the individuals in the combined cohorts that carry the TT genotype at rs7677751 had a 1.65-fold (95%CI = 1.33–2.06, P-value = 6.23×10^−6^) increase in the risk of developing corneal astigmatism compared to those that are not carrying any copies of the risk allele (Figure S4). All of the associated SNPs spanned 10 kb within *PDGFRA* at 4q12 (Figure 2), and a high degree of linkage disequilibrium is seen at this locus in all three Asian populations (Chinese, Malays and Asian Indians; Figure S5).

## Discussion

We have performed a genome-wide survey for corneal astigmatism across 8,513 individuals, where the discovery phase combines the data from two GWAS performed in Chinese and Malay adults, and the replication phase included Asian Indian adults, Chinese children and family trios. We observed a strong and consistent association with the onset of corneal astigmatism at the *PDGFRA* gene locus on chromosome 4q12 across all five Asian cohorts, with three SNPs in this locus exhibiting evidence stronger than genome-wide significance in the meta-analysis. To the best of our knowledge, this is the first GWAS to investigate the genetic etiology of corneal astigmatism in a genome-wide fashion.

The *PDGFRA* gene spans 69 kb with 23 coding exons and resides on chromosome 4q12. The receptor for platelet-derived growth factor (*PDGF*) contains two types of subunit, a- and β- *PDGFRA*, which are differentially expressed on the cell surface [25]. *PDGFR-a* binds to three forms of *PDGF* (*PDGF-AA*, *AB* and BB) and mediates many biological process including embryonic development, angiogenesis, cell proliferation and differentiation. The role of *PDGFRA* in cellular growth and proliferation is underlined by its contribution to the pathogenesis of gastrointestinal stromal tumours [26]. A large body of evidence has shown that both *PDGF* and its receptors are expressed in corneal epithelium, stromal fibroblasts and endothelium [27], [28] as well as proliferative retinal tissues in eyes [29], [30], [31]. Along with other cytokines (epidermal growth factor, transforming growth factor-a,-β etc), studies have further suggested that *PDGF* and its receptors can mediate corneal fibroblast migration, matrix remodeling and play an important role in corneal wound healing [28], [32], [33], [34]. The corneal stroma comprises a large portion of the cornea; the sensitivity of stromal tissue to various growth factors is well described [35]. The administration of *PDGF* resulted in keratinocyte elongation using rabbit corneal stroma tissue [36]. In light of this, a role for *PDGFRA* in the regulation of ocular development and parameters cannot be excluded, and our study suggests that genetic polymorphisms within *PDGFRA* may be involved in the regulation of corneal biometrics resulting in the occurrence of corneal astigmatism.

In addition, Hammond *et al.* reported that 4q12 (MYP9; LOD 3.3) was significant linked with myopia from a genome-wide linkage study of 221 dizygotic twin pairs [24], and subsequent replication revealed nominal significance of 4q12 (*P* = 0.065) for refractive error in African-American families [37]. We thus undertook a candidate SNP approach with the identified SNPs to investigate the possible association between *PDGFRA* and (i) the onset of high myopia; (ii) the refractive error as a quantitative trait. We did not observe any striking association between the identified variants with either outcomes, suggesting that the association of *PDGFRA* with corneal astigmatism is probably not through any shared etiology with myopia.

The lead SNP in our analyses rs7677751 is located in the intro 1 of *PDGFRA*. Interestingly, among the SNPs identified, rs2228230 is coding-synonymous (valine:GTC>valine:GTT) and resides in exon 18, while rs3690 is within the untranslated-3′ region. These three SNPs (rs7677751, rs2228230 and rs3690) are strongly correlated with each other (pair-wise Pearson correlation coefficient r ranging from 0.77 to 0.81), although the association evidence at the latter two SNPs did not reach genome-wide significance. As the next closest gene (*GSX2*) from the 5′ end of *PDGFRA* is 127 kb away and is not within the LD block with our identified SNPs (Figure 1), it is unlikely that the signals observed in our study are attributed to functional variants located beyond *PDGFRA*.

Our group recently reported a strong association between variants in *PDGFRA* with corneal curvature [38]. Corneal curvature is an ocular dimension defined as the average of the radius of corneal curvature at the horizontal and vertical meridians. Myopic eyes have been found to have steeper corneas (reduced radius of curvature), but the significant correlation between corneal curvature and refractive error was not consistently observed [39], [40], [41]. Excessively flatter cornea is associated with cornea plana, producing high hyprotropia and likely resulting in angle-closure glaucoma [42], [43]. On the other hand, corneal astigmatism is an eye-disorder, where the cornea is more curved in one meridian direction compared to the other. This fragmentizes the light rays entering the eye, leading to the inability to focus onto a single point in the eye [1]. It is thus interesting that the same *PDGFRA* gene has been identified in two ocular outcomes that are biologically different, given the presence of a weak correlation between corneal astigmatism and corneal curvature (Spearman correlation coefficient r between 0.088 and 0.192 in our cohorts; Figure S6), pointing to a possible pleiotropic contribution of *PDGFRA*.

Our study has adopted a binary definition of corneal astigmatism (affected and unaffected) that is commonly adopted in clinical practice and eye-trait epidemiology [16], [44]. One caveat of this definition is the potential for misclassifying the affected status, particularly for samples with cylinder power around the cutoff threshold of −0.75D. To evaluate the robustness of our findings to the choice of threshold used, we additionally performed the association analysis at the identified SNPs with four different combinations of the thresholds used to define cases and controls. We observed that the odds ratios were highly similar across all four scenarios (Table S3), with the combined evidence at rs7677751 ranging from *P*_meta_ of 1.5×10^−4^ to 6.7×10^−8^. Unsurprisingly, the association evidence was weakest in the scenario with the most stringent thresholding (≤−1.5D for cases and >−0.5 for controls), given this stringency comes at the expense of decreasing the number of individuals in each study. We additionally performed a secondary analysis treating corneal cylinder power as a quantitative trait. Strong statistical evidence was consistently observed at the three leading SNPs (rs7677751, *P* = 1.76×10^−7^; rs2307049, *P* = 3.41×10^−7^ and rs7660560, *P* = 4.41×10^−7^; Table S4), indicating that our findings are robust to the definition of the phenotype.

Owing to the relatively small sample sizes within each of the five GWAS studies, we have chosen to prioritize our survey to identify genetic variants that contribute to the etiology of corneal astigmatism in multiple Asian populations. While Malays have been observed to be genetically closer to the Chinese, the Asian Indians tend to be genetically closer to the Caucasians [45]. Our discovery at *PDGFRA* thus suggests that part of the underlying biological pathway responsible for astigmatism development is common to multiple populations, although there may be population-specific genetic variants that our current study is not sufficiently powered to identify.

Our study has included two pediatric Chinese populations (SCORM and STARS) with school or pre-school children who are still progressing to their final phenotype. It was documented that a high degree of astigmatism occurs during infancy and the early childhood [46]. The prevalence rates remain stable during young adulthood (18 to 40 years), but increase consistently during late adulthood at aged 40 years or older [1], [12]. Studies have also indicated that the age-related change in astigmatism is associated with meridians changes in the cornea [11]. Children and adolescents have a predominance of “within-the-rule” corneal astigmatism in general, where the vertical curve is greater than the horizontal (axis of 1° to 15°); while in older adults, it shifts to “against-the-rule” astigmatism (axis of 75°–105°) [47], [48]. However, our study considers corneal astigmatism without reference to the axis nor the longitudinal changes from children to adults. Whether *PDGFRA* plays the same role in pediatric and adults populations will however need further investigation.

## Materials and Methods

### Ethics statement

This study adhere to the Declaration of Helsinki. Ethics approvals have been obtained from the Institutional Review Boards of the Singapore Eye Research Institute, Singapore General hospital, National University of Singapore and National Healthcare Group, Singapore. In all cohorts, participants provided written, informed consent at the recruitment into the studies. For studies involving children who were still minors (SCORM and STARS), written informed consent was obtained from the children's parents.

### Discovery cohorts

#### Singapore Prospective Study Program (SP2)

Participants included in SP2 were recruited from a revisit of four previously conducted population-based surveys in Singapore: Thyroid and Heart Study 1982–1984 [49], National Health Survey 1992 [50], National University of Singapore Heart Study 1993–1995 [51] and the National Health Survey 1998 [50], [51], [52]. These studies comprise random samplings of individuals stratified by ethnicity from the entire Singapore population. From 2003 to 2007, a total of 10,747 adults aged 40 years or older were invited in this follow-up survey of which 5,157 underwent a health examination and blood samples were drawn. The present genome-wide genotyping involved individuals of Chinese descent only (n = 2,867). Complete post-filtering data on corneal astigmatism for GWAS were available for 2,016 individuals.

#### Singapore Malay Eye Study (SiMES)

SiMES is a population-based cross-sectional survey on eye diseases for Malay adults aged 40 to 80 years living in Singapore. It was conducted between August of 2004 and June of 2006. The details of the study design and methods have been previously described [53]. In brief, a total of 4,168 Malay residents in the Southwestern area of Singapore were identified and invited for a detailed ocular examination which 3,280 (78.7%) participated. Genome-wide genotyping was performed in 3,072 individuals [54], [55]. Individuals with cataract surgery and missing corneal astigmatism measurements were excluded from the study. Complete post-filtering data on corneal astigmatism for GWAS were available for 2,238 individuals.

### Replication cohorts

#### Singapore Indian Eye Study (SINDI)

SINDI is a population-based survey of major eye diseases [56] in ethnic Indians aged 40 to 80 years living in the South-Western part of Singapore and was conducted from August 2007 to December 2009. In brief, 4,497 Indian adults were eligible and 3,400 participated. Genome-wide genotyping was performed in 2,953 individuals [54], [55]. As in the discovery cohorts, participants were excluded from the study if they had cataract surgery and missing corneal astigmatism data. Complete post-filtering data on corneal astigmatism were available for 2,134 participants.

#### Singapore Cohort Study of the Risk Factors for Myopia (SCORM)

A total of 1,979 children in grades 1, 2, and 3 from three schools were recruited from 1999 to 2001 with detailed information described elsewhere [57]. The children were examined on the school premises annually by a team of eye care professionals. The GWAS was conducted in a subset of Chinese children of 1,116 subjects [58]. The phenotype used in this study was based on the corneal astigmatism measured on the 4^th^ annual examination of the study (children at age 10 to 12 years). Complete post-filtering data on corneal astigmatism measurements and SNP data were available in 929 SCORM children.

#### Singaporean Chinese in the Strabismus, Amblyopia, and Refractive Error Study (STARS)

STARS is a population-based survey of Chinese families with children aged 6 to 72 months residing in the south-western and western region of Singapore. Disproportionate random sampling by 6-month age groups resulted in the recruitment and subsequent eye examination of 3,009 Chinese children between May 2006 and November 2008. Details of the study design and methodology have been previously described [59]. A total of 1,451 samples from 440 nuclear families were included for genome-wide genotyping. In all, 397 trio-sets of parents and their offsprings with corneal astigmatism had complete post-filtered genotype data.

### Measurements and definition of corneal astigmatism

All studies used a similar protocol to measure ocular phenotypes including corneal curvature, autorefraction and cylinder power by a team of eye care professionals. Participants in SP2, SIMES and SINDI underwent non-cycloplegic automated refractive assessments using the autorefractor (Canon RK-5, Tokyo, Japan). For SCORM and STARS, cycloplegic measurements (Canon RK-F1, Tokyo, Japan) were performed 30 minutes after three drops of 1% cyclopentolate which were administered 5 minutes apart.

Corneal curvature radii in the horizontal and vertical meridian were determined with keratometry in millimeters [60]. The keratometer measured the anterior corneal surface and used a refractive index of 1.3375 to account for the contribution from the posterior corneal surface to derive the corneal refractive power in diopters. Corneal cylinder power was calculated as the difference between the flattest and steepest meridian of the keratometry readings in diopters of power.

As the corneal cylinder power between the right and left eyes are strongly correlated across all five cohorts (Pearson's correlation coefficient r ranging from 0.51 to 0.79; P<2.2×10^−16^), the mean corneal cylinder power over both eyes was used to define corneal astigmatism. Averaging ocular measurements between two eyes in genetic studies has been suggested to be statistically more powerful than using the information from only one eye [61], and this approach has been consistently adopted in genome-wide studies of myopia [62], [63]. As with previous studies [16], [44], we have defined individuals with average corneal cylinder power ≤−0.75D as cases, and those with average corneal power between −0.75D and 0D as controls.

### Genotyping and data quality control

For SP2, a total of 2,867 blood-derived DNA samples were genotyped using the Illumina Human610 Quad and 1Mduov3 Beadchips. For the samples that were genotyped on the two platforms, the genotypes from the denser platform were used in our study. For SiMES (n = 3,072), SINDI (n = 2,593) and STARS (n = 1,451), the Illumina Human610 Quad Beadchips was used for genotyping all DNA samples. For the 1,116 SCORM children, DNA samples were genotyped on the Illumina HumanHap 550 Duo Beadchips.

Detailed data quality control (QC) procedures for each study were provided in the supplementary information (Text S1). In brief, for case-control study design, QC criteria included a first round for autosomes SNP QC to obtain a cleaned set of genotypes for sample QC, by excluding SNPs with: (i) missingness (per-SNP call rate) >5%; (ii) minor allele frequency (MAF) <1%; and (iii) HWE p-value<10^−7^. Using the subset of SNPs passing the first round QC, samples were then excluded based on the following conditions: (i) per-sample call rates of less than 95%; (ii) excessive heterozygosity (defined as the sample heterozygosity to be beyond 3 standard deviations from the mean sample heterozygosity); (iii) cryptic relatedness; (iv) gender discrepancies; and (v) deviation in population membership from population structure analysis. A second round of SNP QC was then applied to the remaining samples passing quality checks. We excluded SNPs with missingness >5%, gross departure from HWE (P value<10^−6^), MAF<1% and low concordance between duplicate samples on different genotype platforms (relevant to SP2 samples only).

Population structure was ascertained using principal components analyses (PCA) with the EIGENSTRAT program [64]. Population substructure of SP2 and SiMES was examined by PCA with respect to three population panels in the HapMap samples (Figure S7). Due to the presence of population structure within the Malay and Indian samples (Figures S8 and S9 respectively), we adjusted for the top 5 principal components in the association analyses for the SiMES and SINDI datasets.

For the STARS trios, we additionally excluded samples and trio-sets on the basis of excessive Mendelian inconsistencies defined as having >1% of the post-QC SNPs exhibiting Mendelian errors. SNPs with more than 10% Mendelian errors are excluded from the association analyses, and the genotypes leading to Mendelian errors in all other remaining SNPs are coded as missing. As family trios are more robust to the presence of population structure, we did not exclude any samples due to population structure.

### Statistical analysis

The genome-wide association tests were performed using PLlNK (version 1.07; http://pngu.mgh.harvard.edu/~purcell/plink/) as the primary analysis tool. A logistic regression adjusted for age and gender is used to model the association of genetic markers with corneal astigmatism. For each of SiMES and SINDI, the top 5 principal components of genetic ancestry from the EIGENSTART PCA were also included as covariates to adjust for population stratification in these populations. We assumed an additive genetic model where the genotypes of each SNP is coded as 0, 1, and 2 for the number of minor alleles carried, in keeping with increments in allelic dosage. For family GWAS association tests in STARS, a transmission disequilibrium test (TDT) is used to measure significant distortions in transmission of an allele from heterozygous parents to the affected offspring under the condition of Mendel's law [65].

We also performed a quantitative trait analysis with the average corneal cylinder power as the outcome. This is performed in PLINK for the unrelated samples, and in FBAT (http://www.hsph.harvard.edu/~fbat/fbat.htm) for the family trios. As the distribution of the quantitative trait of corneal cylinder power is skewed (Figure S10), we performed a normal quantile transformation [66] prior to the association analysis for unrelated samples. For family-based data, no transformation was conducted since the FBAT does not require normal trait [67]

Meta-analyses are performed using weighted-inverse variance estimated from fixed-effect modeling in METAL (http://www.sph.umich.edu/csg/abecasis/metal/). We adopt the method by Kazeem and Farrall [65] to pool the evidence from the case-control analyses and the family trio TDT. For the quantitative trait analysis, the overall z statistics is calculated as a weighted sum of the z-statistics from the linear regressions in the non-familial data and FBAT analysis for the family-based data, weighted by number of unrelated individuals or trios in the respective studies [68].

Results from a genome-wide meta-analysis of the SNPs common to SP2 and SiMES are used in the discovery phase to identify putative variants that are associated with the onset of corneal astigmatism, defined as a *P*-value<10^−5^. The remaining three cohorts (SINDI, SCORM and STARS) are used to validate the putative findings. In addition, a genome-wide meta-analysis of all five datasets is also performed. Genotyping quality of all reported SNPs in this paper have been visually assessed by checking the intensity clusterplots.

## Supporting Information

Figure S1 Quantile-Quantile (Q-Q) plots of *P*-values for association between all SNPs and corneal astigmatism in the combined meta-analysis of the discovery cohorts (A) individual cohort SP2, (B) SiMES, and (C) SP2+SiMES.
(TIF)

Figure S2 (A) Manhattan plot of log_10_(P-values) in the combined discovery cohort of SP2 and SiMES. The blue horizontal line presents the threshold of suggestive significance (*P* = 1.00×10^−5^). (B) Regional SNP association plot for the corneal astigmatism (≤−0.75D) by the association scatter plot for SNPs in the *PDGFRA* gene in the combined meta-analysis for discovery cohort SP2+SiMES.
(TIF)

Figure S3 (A) Quantile-Quantile (Q-Q) plot of *P*-values for association between all SNPs and corneal astigmatism in the combined meta-analysis of the fiver cohorts SP2, SiMES, SINDI, SCORM and STARS. (B) Manhattan plots of *P*-values for the association on corneal astigmatism in the meta-analysis of five cohorts.
(TIF)

Figure S4 Forest plots of association of homozygotes TT of rs7677751 for corneal stigmatism (≤−0.75 D). Odds ratios for individuals carrying two copies of the risk allele T of rs7677751 are estimated for the five Asian populations. Homozygous odds ratio for family-base data (STARS) is calculated based on previously described method [69].
(TIF)

Figure S5 Linkage disequilibrium (LD) calculated in terms of D' for Singapore Chinese samples from SP2 (A), Malays samples from SiMES (B) and Indians panels from SINID (C). Black squares show perfect LD whereas shades of grey show decreasing LD.
(TIF)

Figure S6 Scatter plots of corneal cylinder power in diopters (D) (quantitative measurements of corneal stigmatism) versus corneal curvature in millimeter (mm) among the common datasets consisted of these two phenotypes. (A) SP2 (n = 2,010; Spearman correlation coefficient r = 0.145; p = 7.57×10^−11^), (B) SiMES (n = 2,237, r = 0.076, p = 3.14×10^−4^), (C) SINDI (n = 2,139, r = 0.088; p = 4.63×10^−5^), (D) SCORM (n = 929, r = 0.192; p = 3.34×10^−9^). Fitted line is predicated from the linear regression by regressing corneal curvature on corneal cylinder power values.
(TIF)

Figure S7 Principal component analysis (PCA) of discovery cohort SP2 and SiMES with respect to the population panels in phase 2 of the HapMap samples (CEU - European, YRI – African, CHB – Chinese, JPT – Japanese). (A) 1^st^ eigenvector against 2^nd^ eigenvector, (B) 2^nd^ eigenvector against 3^rd^ eigenvector.
(TIF)

Figure S8 Principal component analysis (PCA) was performed in SiMES to assess the extent of population structure. Each figure represents a bivariate plot of two principal components from the PCA analysis of genetic diversity within SiMES. (A) 1^st^ eigenvector against 2^nd^ eigenvector, (B) 2^nd^ eigenvector against 3^rd^ eigenvector, (C) 3^rd^ eigenvector against 4^th^ eigenvector and (D) 1^nd^ eigenvector against 5^th^ eigenvector. The first 5 principal components were used as covariates to account for population structure.
(TIF)

Figure S9 Principal component analysis (PCA) was performed in SINDI to assess the extent of population structure. Each figure represents a bivariate plot of two principal components from the PCA analysis of genetic diversity within SINDI. (A) 1^st^ eigenvector against 2^nd^ eigenvector, (B) 2^nd^ eigenvector against 3^rd^ eigenvector, (C) 3^rd^ eigenvector against 4^th^ eigenvector and (D) 1^nd^ eigenvector against 5^th^ eigenvector. The first 5 principal components were used as covariates to account for population structure.
(TIF)

Figure S10 Histogram of average corneal cylinder power and the normal transformed values for non-familial cohorts: SP2, SiMES, SINDI, and SCORM. The values of corneal cylinder power were transformed by a normal quantile transformation [66] and used in the association tests.
(TIF)

Table S1 Minor allele frequencies (MAFs) of the top SNPs across different populations.
(DOCX)

Table S2 Top SNPs (*P*-value≤5×10^−6^) identified from combined meta-analysis of five Asian population cohorts.
(DOCX)

Table S3 The association results in the five cohorts and combined meta-analysis under different scenarios with varying threshold to define the cases and controls for corneal astigmatism. Cases are defined as corneal cylinder power ≤−1.0D in setting (A) and (B), while controls are defined as corneal cylinder power >−0.75D(A) or >−0.5D (B) in case-control design. For family-based STARS cohort in setting (A) and (B), we performed TDT on those families with corneal astigmatic children only (≤−1.0D). Similarly, cases are defined as corneal cylinder power ≤−1.5D in setting (C) and (D), while controls are defined as corneal cylinder power >−0.75D(C) or >−0.5D (D) in case-control design. For STARS cohort in setting (C) and (D), we performed TDT on those families with corneal astigmatic children only (≤−1.5D).
(DOCX)

Table S4 Top SNPs (*P*-value≤1×10^−5^) identified from the meta-analysis of five GWAS with corneal astigmatism as a quantitative trait. A*: effect allele; no allelic effect size (β) estimated for STARS (parent-trios design) using FBAT on family-based quantitative trait.
(DOCX)

Text S1 Detailed QC procedures for SP2, SiMES, SINDI, SCORM and STARS.
(DOCX)
