# Supplementary figures and images for: Genome-Wide Meta-Analysis of Five Asian Cohorts Identifies PDGFRA as a Susceptibility Locus for Corneal Astigmatism
Source: PLoS Genet. 2011 Dec 1;7(12):e1002402. doi: 10.1371/journal.pgen.1002402 (PMC3228826; doi:10.1371/journal.pgen.1002402)

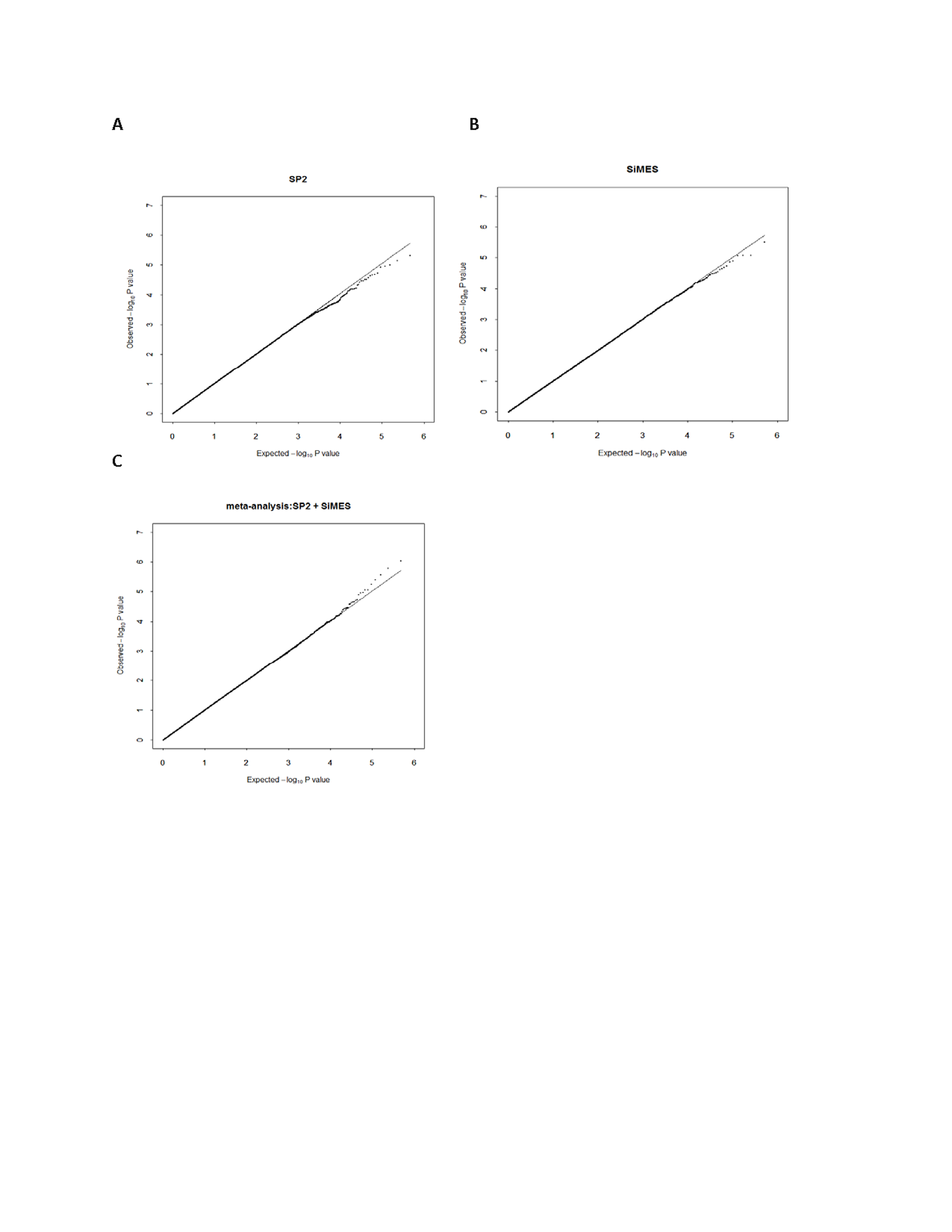

Supplement: Figure S1 — Quantile-Quantile (Q-Q) plots of P-values for association between all SNPs and corneal astigmatism in the combined meta-analysis of the discovery cohorts (A) individual cohort SP2, (B) SiMES, and (C) SP2+SiMES. (TIF) [file pgen.1002402.s001.tif]

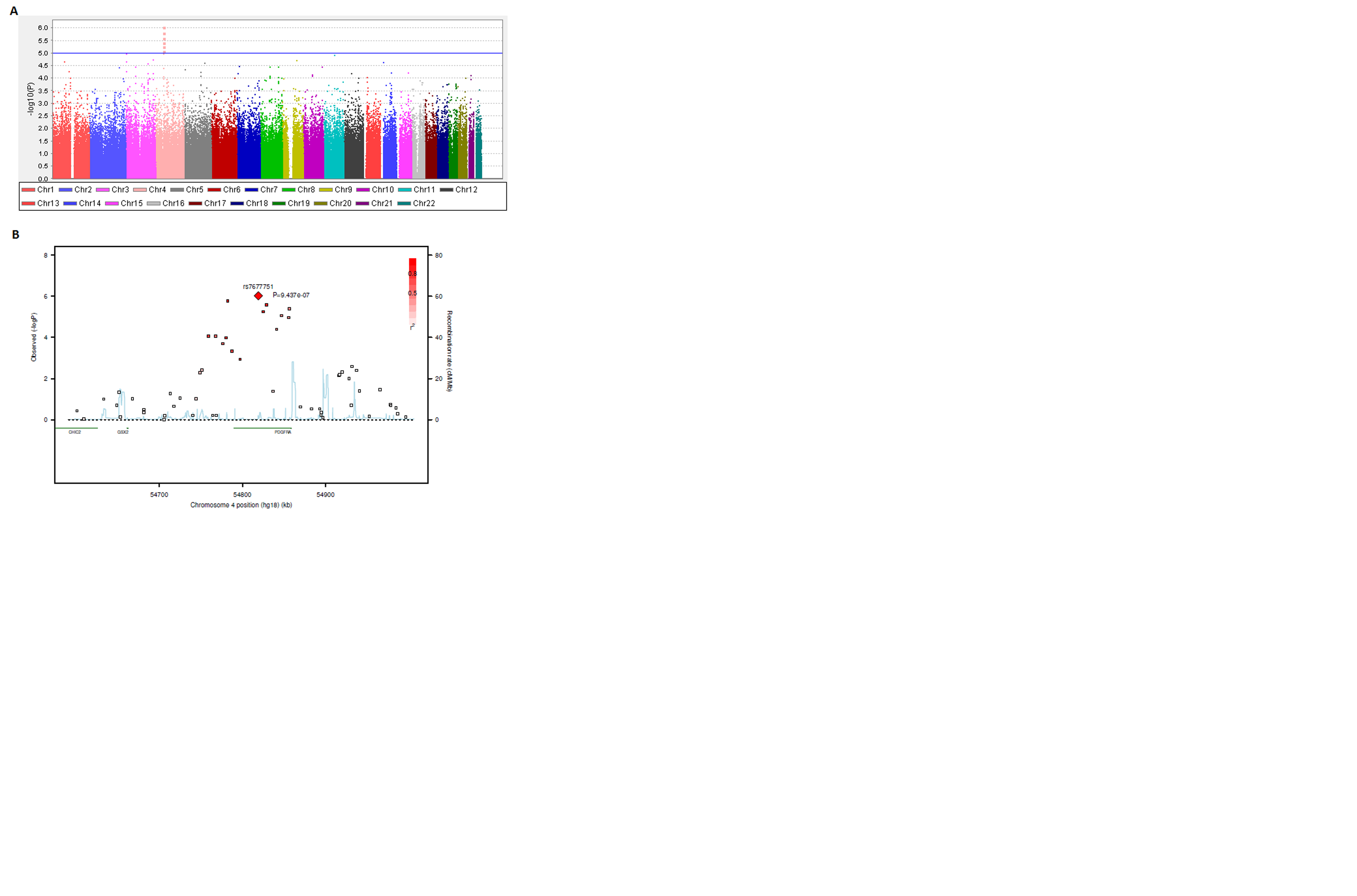

Supplement: Figure S2 — (A) Manhattan plot of log10(P-values) in the combined discovery cohort of SP2 and SiMES. The blue horizontal line presents the threshold of suggestive significance (P = 1.00×10^−5^). (B) Regional SNP association plot for the corneal astigmatism (≤−0.75D) by the association scatter plot for SNPs in the PDGFRA gene in the combined meta-analysis for discovery cohort SP2+SiMES. (TIF) [file pgen.1002402.s002.tif]

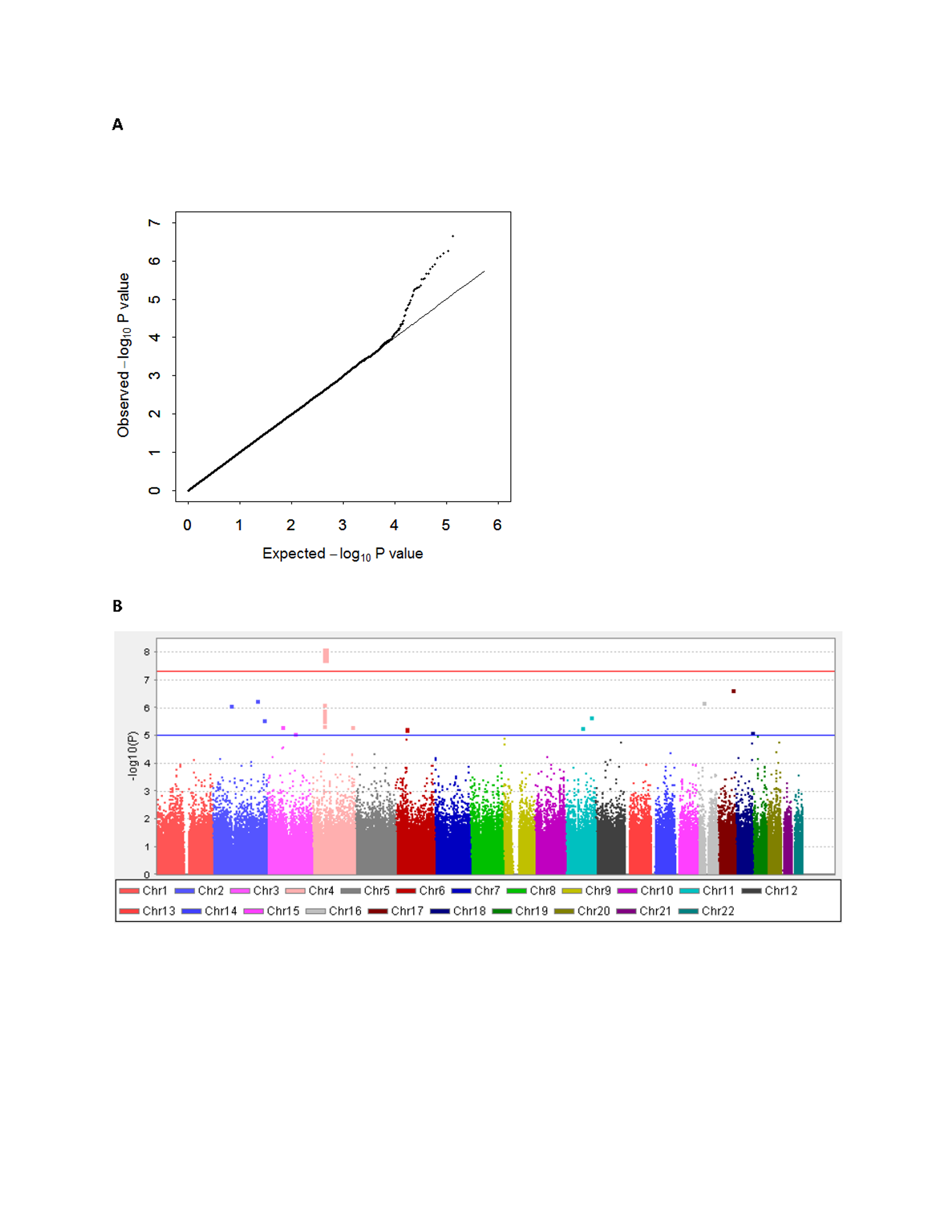

Supplement: Figure S3 — (A) Quantile-Quantile (Q-Q) plot of P-values for association between all SNPs and corneal astigmatism in the combined meta-analysis of the fiver cohorts SP2, SiMES, SINDI, SCORM and STARS. (B) Manhattan plots of P-values for the association on corneal astigmatism in the meta-analysis of five cohorts. (TIF) [file pgen.1002402.s003.tif]

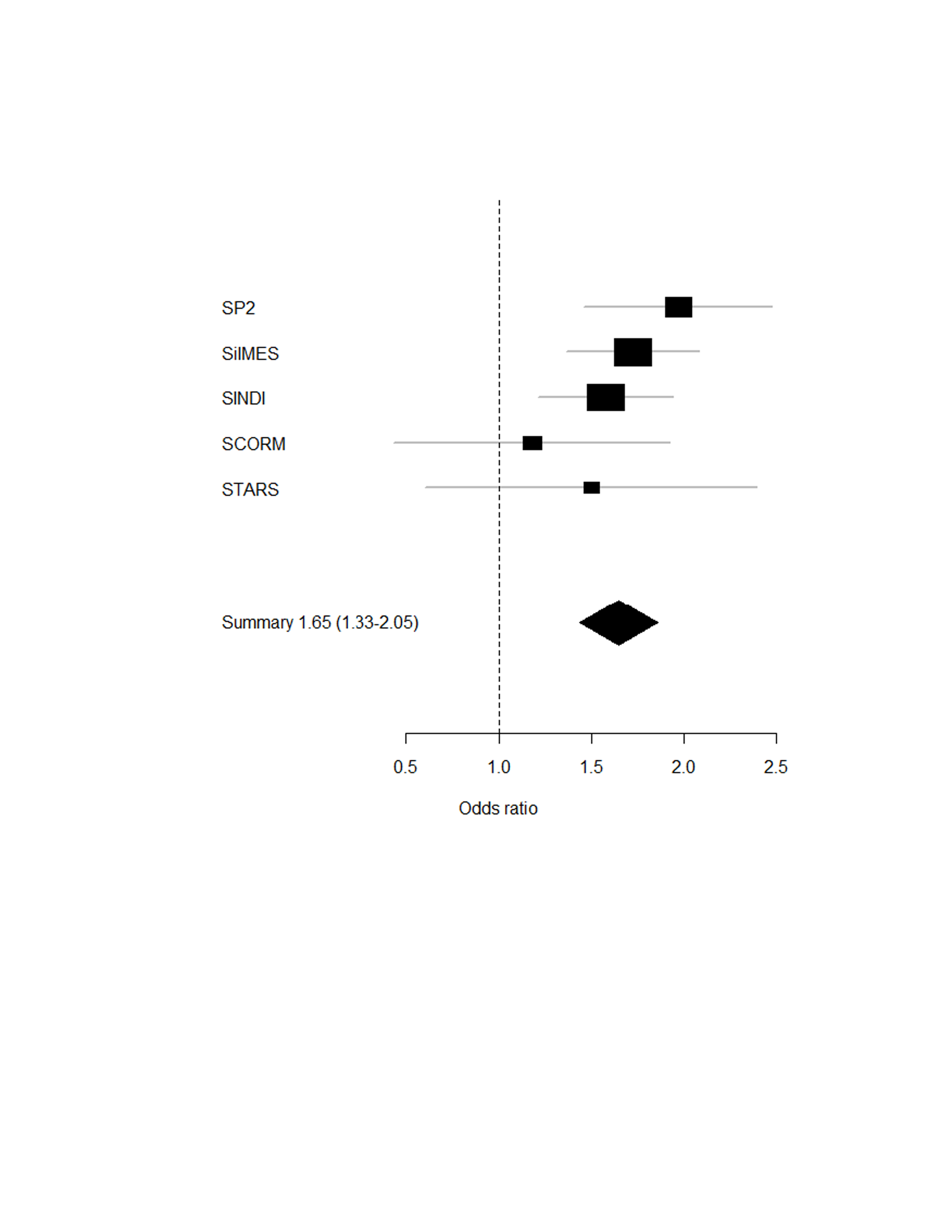

Supplement: Figure S4 — Forest plots of association of homozygotes TT of rs7677751 for corneal stigmatism (≤−0.75 D). Odds ratios for individuals carrying two copies of the risk allele T of rs7677751 are estimated for the five Asian populations. Homozygous odds ratio for family-base data (STARS) is calculated based on previously described method [69]. (TIF) [file pgen.1002402.s004.tif]

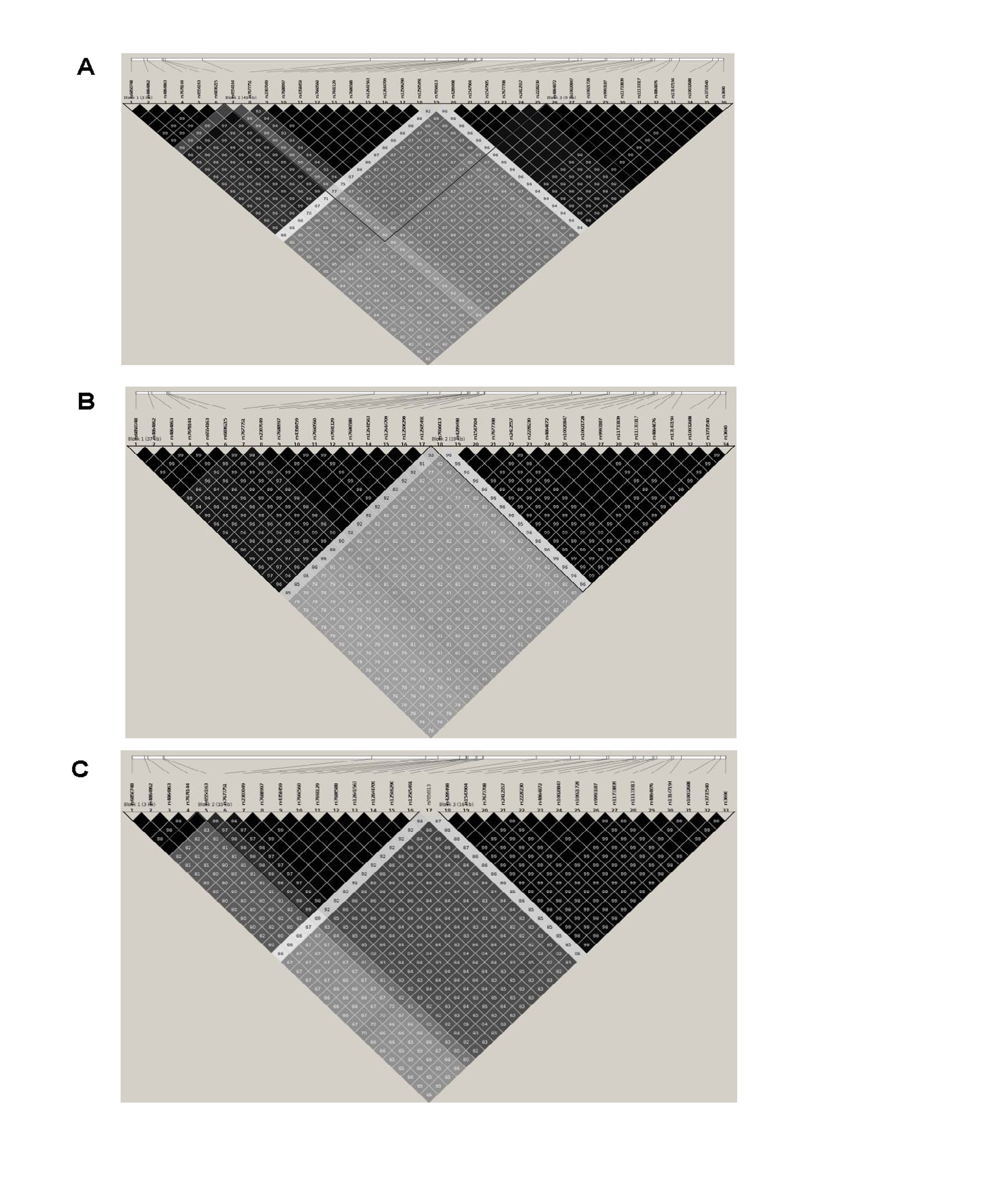

Supplement: Figure S5 — Linkage disequilibrium (LD) calculated in terms of D' for Singapore Chinese samples from SP2 (A), Malays samples from SiMES (B) and Indians panels from SINID (C). Black squares show perfect LD whereas shades of grey show decreasing LD. (TIF) [file pgen.1002402.s005.tif]

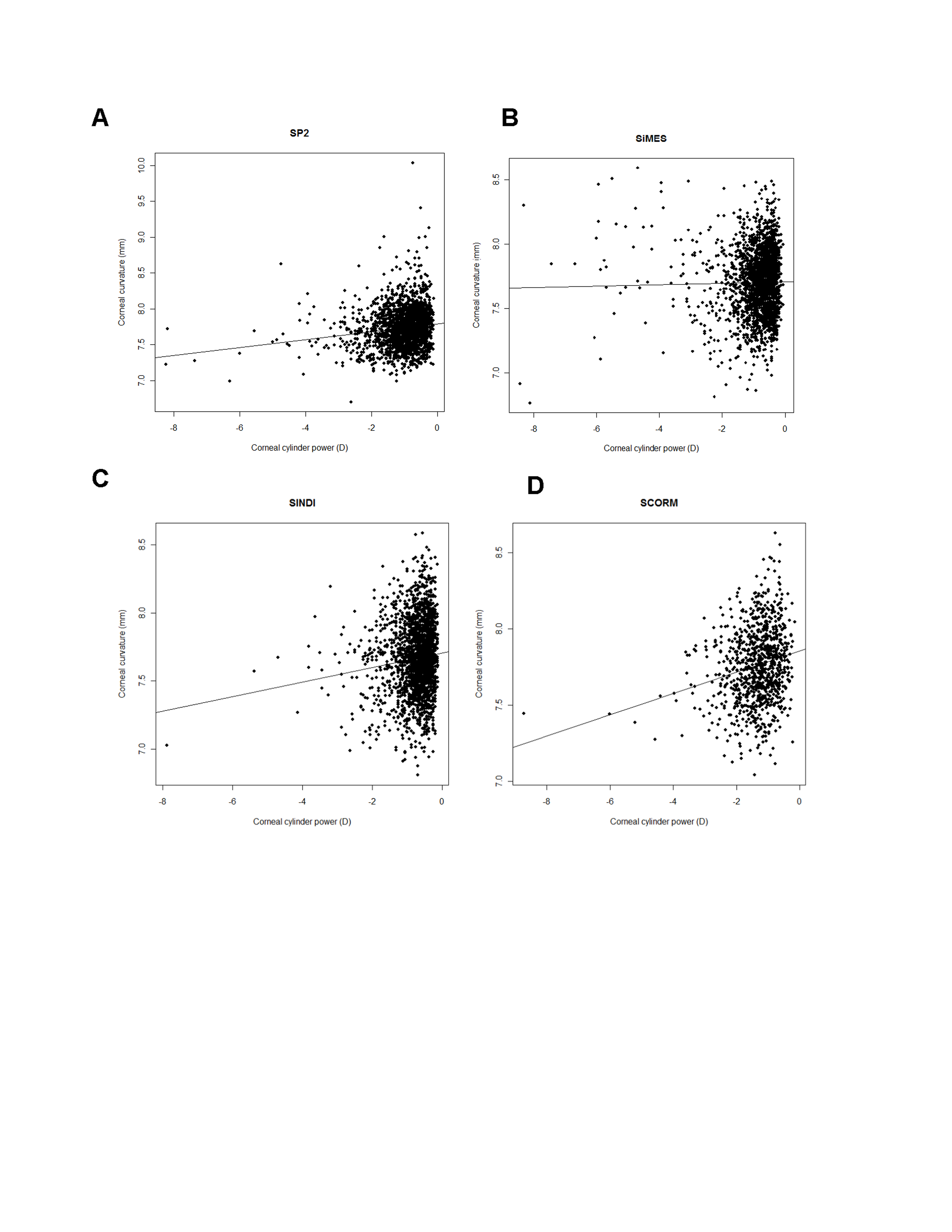

Supplement: Figure S6 — Scatter plots of corneal cylinder power in diopters (D) (quantitative measurements of corneal stigmatism) versus corneal curvature in millimeter (mm) among the common datasets consisted of these two phenotypes. (A) SP2 (n = 2,010; Spearman correlation coefficient r = 0.145; p = 7.57×10^−11^), (B) SiMES (n = 2,237, r = 0.076, p = 3.14×10^−4^), (C) SINDI (n = 2,139, r = 0.088; p = 4.63×10^−5^), (D) SCORM (n = 929, r = 0.192; p = 3.34×10^−9^). Fitted line is predicated from the linear regression by regressing corneal curvature on corneal cylinder power values. (TIF) [file pgen.1002402.s006.tif]

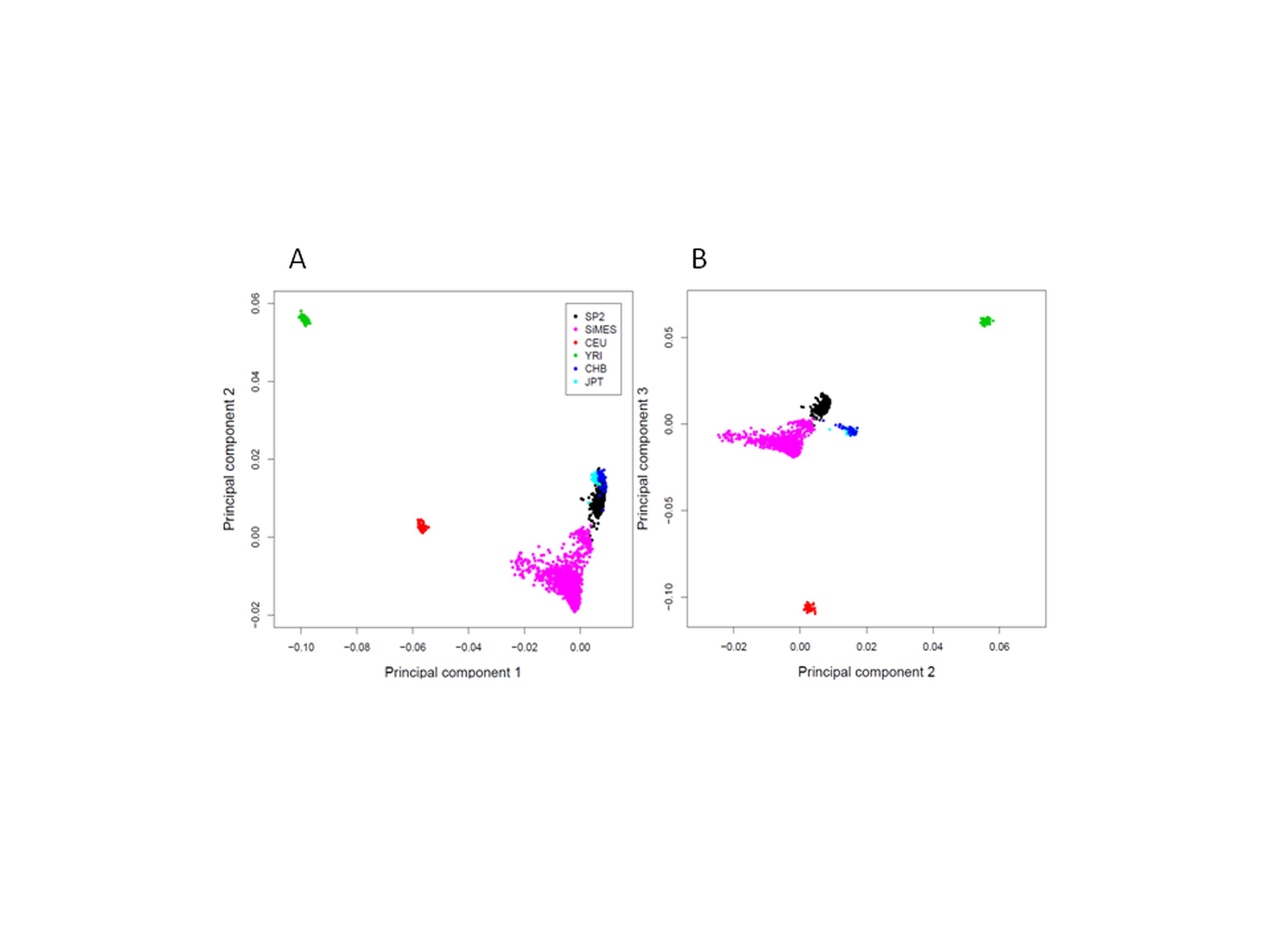

Supplement: Figure S7 — Principal component analysis (PCA) of discovery cohort SP2 and SiMES with respect to the population panels in phase 2 of the HapMap samples (CEU - European, YRI – African, CHB – Chinese, JPT – Japanese). (A) 1st eigenvector against 2nd eigenvector, (B) 2nd eigenvector against 3rd eigenvector. (TIF) [file pgen.1002402.s007.tif]

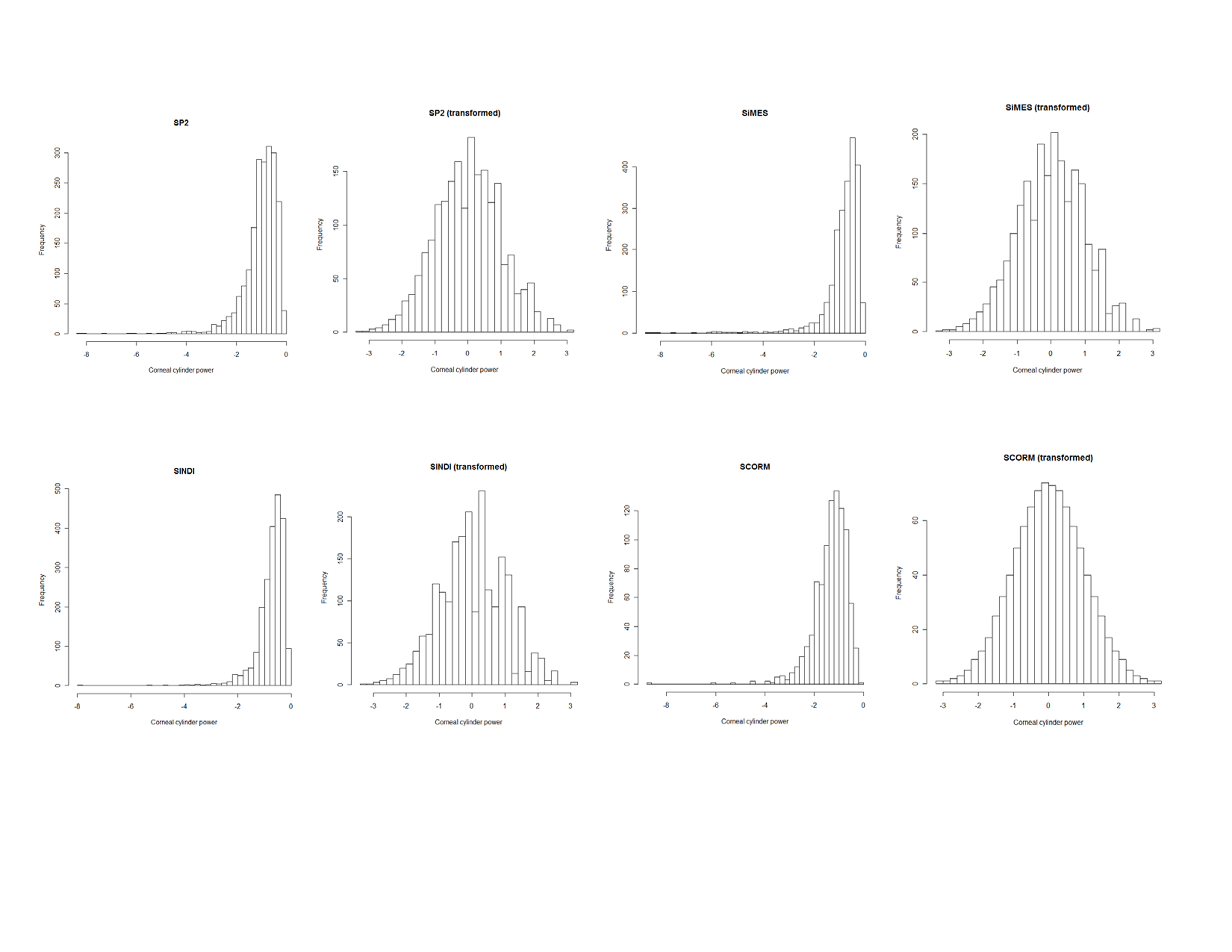

Supplement: Figure S10 — Histogram of average corneal cylinder power and the normal transformed values for non-familial cohorts: SP2, SiMES, SINDI, and SCORM. The values of corneal cylinder power were transformed by a normal quantile transformation [66] and used in the association tests. (TIF) [file pgen.1002402.s010.tif]
