# Supplementary material for: Genome-Wide Meta-Analysis of Five Asian Cohorts Identifies PDGFRA as a Susceptibility Locus for Corneal Astigmatism
Source: PLoS Genet. 2011 Dec 1;7(12):e1002402. doi: 10.1371/journal.pgen.1002402 (PMC3228826; doi:10.1371/journal.pgen.1002402)
Supplement: Table S1 — Minor allele frequencies (MAFs) of the top SNPs across different populations. (DOCX) [file pgen.1002402.s011.docx]

**Table S1**

|  | |  |  |  |  | MAF | | | | |
| --- | --- | --- | --- | --- | --- | --- | --- | --- | --- | --- |
| CHR | SNP | | BP | A1 | A2 | SP2 | SiMES | SINDI | SCORM | STARS |
| 4 | rs17084051 | | 54782338 | A | C | 0.21 | 0.25 | 0.26 | 0.20 | 0.20 |
| 4 | rs7677751 | | 54819217 | T | C | 0.19 | 0.25 | 0.26 | 0.19 | 0.19 |
| 4 | rs2307049 | | 54824911 | A | G | 0.18 | 0.25 | 0.26 | 0.18 | 0.18 |
| 4 | rs7660560 | | 54829151 | A | G | 0.18 | 0.25 | 0.26 | 0.19 | 0.18 |
| 4 | rs2228230 | | 54846797 | T | C | 0.14 | 0.17 | 0.26 | 0.14 | 0.15 |
| 4 | rs4864872 | | 54847041 | T | G | 0.14 | 0.17 | 0.26 | 0.14 | 0.15 |
| 4 | rs3690 | | 54856570 | C | A | 0.14 | 0.17 | 0.26 | 0.14 | 0.15 |

A1: minor allele; A2: major allele.
