# Supplementary material for: Genome-Wide Meta-Analysis of Five Asian Cohorts Identifies PDGFRA as a Susceptibility Locus for Corneal Astigmatism
Source: PLoS Genet. 2011 Dec 1;7(12):e1002402. doi: 10.1371/journal.pgen.1002402 (PMC3228826; doi:10.1371/journal.pgen.1002402)
Supplement: Table S2 — Top SNPs (P-value≤5×10^−6^) identified from combined meta-analysis of five Asian population cohorts. (DOCX) [file pgen.1002402.s012.docx]

**Table S2**

|  |  |  |  |  | **SP2** | | | **SiMES** | | | **SINDI** | | | **SCORM** | | |  | **STARS** | |  |  | **Meta-analysis** | |
| --- | --- | --- | --- | --- | --- | --- | --- | --- | --- | --- | --- | --- | --- | --- | --- | --- | --- | --- | --- | --- | --- | --- | --- |
| **SNP** | **GENE** | **CHR** | **BP** | **A*** | **OR** | **s.e.** | **P** | **OR** | **s.e.** | **P** | **OR** | **s.e.** | **P** | **OR** | **s.e.** | **P** | **OR** | **s.e.** | **P** | | **OR** | **s.e.** | **P** |
| rs7677751 | PDGFRA | 4 | 54819217 | T | 1.35 | 0.09 | 3.76E-04 | 1.27 | 0.07 | 6.26E-04 | 1.23 | 0.07 | 4.09E-03 | 1.22 | 0.15 | 2.00E-01 | 1.12 | 0.13 | 3.74E-01 | | 1.30 | 0.05 | 7.87E-09 |
| rs7660560 | PDGFRA | 4 | 54829151 | A | 1.32 | 0.09 | 1.46E-03 | 1.28 | 0.07 | 5.34E-04 | 1.26 | 0.07 | 1.37E-03 | 1.13 | 0.15 | 4.22E-01 | 1.14 | 0.13 | 3.20E-01 | | 1.29 | 0.05 | 1.15E-08 |
| rs2307049 | PDGFRA | 4 | 54824911 | A | 1.31 | 0.09 | 2.17E-03 | 1.26 | 0.07 | 7.55E-04 | 1.27 | 0.07 | 1.08E-03 | 1.14 | 0.15 | 4.03E-01 | 1.14 | 0.13 | 3.17E-01 | | 1.28 | 0.05 | 1.58E-08 |
| rs10189905 |  | 2 | 199387355 | G | 0.72 | 0.11 | 2.56E-03 | 0.78 | 0.08 | 4.13E-03 | 0.83 | 0.12 | 1.09E-01 | 1.08 | 0.21 | 7.05E-01 | 0.61 | 0.18 | 5.70E-03 | | 0.76 | 0.07 | 5.57E-07 |
| rs3690 | PDGFRA | 4 | 54856570 | C | 1.40 | 0.10 | 6.25E-04 | 1.29 | 0.08 | 1.53E-03 | 1.18 | 0.07 | 2.17E-02 | 1.03 | 0.17 | 8.58E-01 | 1.09 | 0.14 | 5.28E-01 | | 1.33 | 0.06 | 7.77E-07 |
| rs4864872 | PDGFRA | 4 | 54847041 | T | 1.41 | 0.10 | 5.94E-04 | 1.26 | 0.08 | 3.19E-03 | 1.19 | 0.07 | 1.49E-02 | 1.05 | 0.17 | 7.69E-01 | 1.06 | 0.14 | 6.71E-01 | | 1.32 | 0.06 | 1.24E-06 |
| rs2228230 | PDGFRA | 4 | 54846797 | T | 1.41 | 0.10 | 5.94E-04 | 1.26 | 0.08 | 3.19E-03 | 1.19 | 0.07 | 1.67E-02 | 1.05 | 0.17 | 7.69E-01 | 1.06 | 0.14 | 6.71E-01 | | 1.32 | 0.06 | 1.43E-06 |
| rs10032688 |  | 4 | 54855415 | A | 1.39 | 0.10 | 8.28E-04 | 1.26 | 0.08 | 3.07E-03 | 1.20 | 0.07 | 1.39E-02 | 1.02 | 0.17 | 8.97E-01 | 1.11 | 0.14 | 4.80E-01 | | 1.31 | 0.06 | 1.64E-06 |
| rs17084051 |  | 4 | 54782338 | A | 1.35 | 0.08 | 3.68E-04 | 1.26 | 0.07 | 1.08E-03 | 1.14 | 0.07 | 7.77E-02 | 1.23 | 0.15 | 1.68E-01 | 1.04 | 0.12 | 7.55E-01 | | 1.29 | 0.05 | 2.16E-06 |
| rs6758183 |  | 2 | 226709230 | A | 1.37 | 0.14 | 2.36E-02 | 1.41 | 0.11 | 1.58E-03 | 1.21 | 0.07 | 1.10E-02 | 1.17 | 0.25 | 5.24E-01 | 1.33 | 0.21 | 1.78E-01 | | 1.39 | 0.09 | 2.80E-06 |
| rs7676985 |  | 4 | 54759330 | A | 1.26 | 0.08 | 2.41E-03 | 1.19 | 0.07 | 1.10E-02 | 1.14 | 0.07 | 6.09E-02 | 1.36 | 0.15 | 3.82E-02 | 1.09 | 0.12 | 4.51E-01 | | 1.22 | 0.05 | 2.98E-06 |
| rs1547904 | PDGFRA | 4 | 54841146 | T | 1.35 | 0.10 | 1.77E-03 | 1.24 | 0.08 | 6.10E-03 | 1.19 | 0.07 | 1.52E-02 | 1.09 | 0.17 | 6.19E-01 | 1.07 | 0.14 | 6.27E-01 | | 1.28 | 0.06 | 4.41E-06 |
| rs6792584 | SUCLG2 | 3 | 67614078 | G | 1.10 | 0.08 | 2.61E-01 | 1.36 | 0.07 | 8.33E-06 | 1.16 | 0.07 | 4.06E-02 | 1.02 | 0.14 | 8.72E-01 | 1.17 | 0.13 | 2.21E-01 | | 1.24 | 0.05 | 4.72E-06 |
| rs2270676 | KCNE3 | 11 | 73846059 | G | 0.74 | 0.10 | 1.89E-03 | 0.80 | 0.07 | 1.67E-03 | 0.90 | 0.08 | 1.76E-01 | 1.08 | 0.18 | 6.75E-01 | 0.80 | 0.14 | 1.18E-01 | | 1.29 | 0.06 | 5.09E-06 |
