# Supplementary material for: Genome-Wide Meta-Analysis of Five Asian Cohorts Identifies PDGFRA as a Susceptibility Locus for Corneal Astigmatism
Source: PLoS Genet. 2011 Dec 1;7(12):e1002402. doi: 10.1371/journal.pgen.1002402 (PMC3228826; doi:10.1371/journal.pgen.1002402)
Supplement: Table S3 — The association results in the five cohorts and combined meta-analysis under different scenarios with varying threshold to define the cases and controls for corneal astigmatism. Cases are defined as corneal cylinder power ≤−1.0D in setting (A) and (B), while controls are defined as corneal cylinder power >−0.75D(A) or >−0.5D (B) in case-control design. For family-based STARS cohort in setting (A) and (B), we performed TDT on those families with corneal astigmatic children only (≤−1.0D). Similarly, cases are defined as corneal cylinder power ≤−1.5D in setting (C) and (D), while controls are defined as corneal cylinder power >−0.75D(C) or >−0.5D (D) in case-control design. For STARS cohort in setting (C) and (D), we performed TDT on those families with corneal astigmatic children only (≤−1.5D). (DOCX) [file pgen.1002402.s013.docx]

**Table S3**

|  |  |  |  |  | **SP2** |  |  | **SiMES** | |  | **SINDI** | |  | **SCORM** | | |  | **STARS** | | **Meta-analysis** | | |
| --- | --- | --- | --- | --- | --- | --- | --- | --- | --- | --- | --- | --- | --- | --- | --- | --- | --- | --- | --- | --- | --- | --- |
| **# Cases/# Controls** | | |  |  | **862/785** | |  | **629/1220** | |  | **462/1314** | |  | **618/169** | | |  | **348 trios** | |  |  |  |
|  | **SNP** | **BP** | **A1** | **OR** | **s.e** | ***P*** | **OR** | **s.e** | ***P*** | **OR** | **s.e** | ***P*** | **OR** | **s.e** | | ***P*** | **OR** | **s.e** | ***P*** | **OR** | **s.e** | ***P*** |
| **A** | rs17084051 | 54782338 | A | 1.34 | 0.09 | 1.23E-03 | 1.27 | 0.08 | 3.31E-03 | 1.25 | 0.09 | 8.84E-03 | 1.24 | 0.15 | 1.57E-01 | | 1.14 | 0.13 | 3.17E-01 | 1.26 | 0.04 | 1.25E-07 |
|  | rs7677751 | 54819217 | T | 1.35 | 0.09 | 1.09E-03 | 1.26 | 0.08 | 3.78E-03 | 1.26 | 0.09 | 7.81E-03 | 1.22 | 0.15 | 2.00E-01 | | 1.21 | 0.14 | 1.54E-01 | 1.27 | 0.04 | 6.66E-08 |
|  | rs2307049 | 54824911 | A | 1.30 | 0.09 | 5.58E-03 | 1.26 | 0.08 | 3.50E-03 | 1.30 | 0.09 | 2.59E-03 | 1.15 | 0.15 | 3.64E-01 | | 1.25 | 0.14 | 1.18E-01 | 1.27 | 0.05 | 1.21E-07 |
|  | rs7660560 | 54829151 | A | 1.32 | 0.09 | 3.61E-03 | 1.27 | 0.08 | 2.53E-03 | 1.29 | 0.09 | 3.96E-03 | 1.14 | 0.15 | 3.89E-01 | | 1.25 | 0.14 | 1.20E-01 | 1.27 | 0.05 | 9.95E-08 |
|  | rs2228230 | 54846797 | T | 1.41 | 0.11 | 1.36E-03 | 1.22 | 0.09 | 3.30E-02 | 1.23 | 0.09 | 1.90E-02 | 1.04 | 0.17 | 8.07E-01 | | 1.06 | 0.15 | 7.07E-01 | 1.22 | 0.05 | 3.79E-05 |
|  | rs4864872 | 54847041 | T | 1.41 | 0.11 | 1.36E-03 | 1.22 | 0.09 | 3.30E-02 | 1.23 | 0.09 | 1.64E-02 | 1.04 | 0.17 | 8.07E-01 | | 1.06 | 0.15 | 7.07E-01 | 1.23 | 0.05 | 3.30E-05 |
|  | rs3690 | 54856570 | C | 1.40 | 0.11 | 1.40E-03 | 1.25 | 0.09 | 1.36E-02 | 1.22 | 0.09 | 2.40E-02 | 1.02 | 0.17 | 8.90E-01 | | 1.08 | 0.15 | 6.01E-01 | 1.23 | 0.05 | 1.98E-05 |
|  |  |  |  |  |  |  |  |  |  |  |  |  |  |  |  | |  |  |  |  |  |  |
|  | |  |  |  | **862/437** | |  | **629/773** | |  | **462/831** | |  | **618/49** | | |  | **348 trios** | |  |  |  |
| **B** | rs17084051 | 54782338 | A | 1.34 | 0.11 | 7.37E-03 | 1.26 | 0.09 | 8.05E-03 | 1.27 | 0.09 | 1.01E-02 | 1.34 | 0.27 | 2.83E-01 | | 1.14 | 0.13 | 3.17E-01 | 1.27 | 0.05 | 2.59E-06 |
|  | rs7677751 | 54819217 | T | 1.35 | 0.11 | 7.43E-03 | 1.27 | 0.09 | 7.77E-03 | 1.26 | 0.09 | 1.44E-02 | 1.32 | 0.27 | 3.16E-01 | | 1.21 | 0.14 | 1.54E-01 | 1.28 | 0.05 | 1.74E-06 |
|  | rs2307049 | 54824911 | A | 1.30 | 0.11 | 2.37E-02 | 1.26 | 0.09 | 9.04E-03 | 1.28 | 0.09 | 8.24E-03 | 1.12 | 0.26 | 6.63E-01 | | 1.25 | 0.14 | 1.18E-01 | 1.27 | 0.05 | 3.95E-06 |
|  | rs7660560 | 54829151 | A | 1.31 | 0.11 | 1.91E-02 | 1.27 | 0.09 | 7.66E-03 | 1.26 | 0.09 | 1.27E-02 | 1.13 | 0.26 | 6.48E-01 | | 1.25 | 0.14 | 1.20E-01 | 1.26 | 0.05 | 4.30E-06 |
|  | rs2228230 | 54846797 | T | 1.41 | 0.13 | 8.12E-03 | 1.26 | 0.10 | 2.28E-02 | 1.21 | 0.09 | 4.36E-02 | 1.35 | 0.33 | 3.73E-01 | | 1.06 | 0.15 | 7.07E-01 | 1.24 | 0.06 | 1.12E-04 |
|  | rs4864872 | 54847041 | T | 1.41 | 0.13 | 8.12E-03 | 1.26 | 0.10 | 2.28E-02 | 1.21 | 0.09 | 3.86E-02 | 1.35 | 0.33 | 3.73E-01 | | 1.06 | 0.15 | 7.07E-01 | 1.24 | 0.06 | 1.01E-04 |
|  | rs3690 | 54856570 | C | 1.41 | 0.13 | 8.29E-03 | 1.30 | 0.10 | 1.06E-02 | 1.20 | 0.09 | 5.42E-02 | 1.35 | 0.33 | 3.66E-01 | | 1.08 | 0.15 | 6.01E-01 | 1.25 | 0.06 | 5.96E-05 |
|  |  |  |  |  |  |  |  |  |  |  |  |  |  |  |  | |  |  |  |  |  |  |
|  |  |  |  |  | **355/785** | |  | **232/1220** | |  | **159/1314** | |  | **300/169** | | |  | **222 trios** | |  |  |  |
| **C** | rs17084051 | 54782338 | A | 1.40 | 0.11 | 3.13E-03 | 1.31 | 0.12 | 1.98E-02 | 1.10 | 0.14 | 4.71E-01 | 1.22 | 0.17 | 2.32E-01 | | 1.06 | 0.17 | 7.32E-01 | 1.25 | 0.06 | 2.72E-04 |
|  | rs7677751 | 54819217 | T | 1.54 | 0.12 | 2.19E-04 | 1.31 | 0.12 | 2.09E-02 | 1.12 | 0.14 | 4.17E-01 | 1.22 | 0.17 | 2.35E-01 | | 1.13 | 0.17 | 4.86E-01 | 1.29 | 0.06 | 2.89E-05 |
|  | rs2307049 | 54824911 | A | 1.47 | 0.12 | 1.33E-03 | 1.31 | 0.12 | 1.91E-02 | 1.13 | 0.14 | 3.59E-01 | 1.13 | 0.17 | 4.82E-01 | | 1.14 | 0.18 | 4.69E-01 | 1.26 | 0.06 | 1.35E-04 |
|  | rs7660560 | 54829151 | A | 1.48 | 0.12 | 1.02E-03 | 1.32 | 0.12 | 1.64E-02 | 1.12 | 0.14 | 4.26E-01 | 1.12 | 0.17 | 5.09E-01 | | 1.16 | 0.18 | 4.17E-01 | 1.27 | 0.06 | 1.22E-04 |
|  | rs2228230 | 54846797 | T | 1.45 | 0.14 | 6.40E-03 | 1.33 | 0.13 | 2.83E-02 | 1.13 | 0.14 | 3.62E-01 | 1.11 | 0.19 | 5.79E-01 | | 1.00 | 0.19 | 1.00E+00 | 1.23 | 0.07 | 1.82E-03 |
|  | rs4864872 | 54847041 | T | 1.45 | 0.14 | 6.40E-03 | 1.33 | 0.13 | 2.83E-02 | 1.13 | 0.14 | 3.62E-01 | 1.11 | 0.19 | 5.79E-01 | | 1.00 | 0.19 | 1.00E+00 | 1.23 | 0.07 | 1.82E-03 |
|  | rs3690 | 54856570 | C | 1.44 | 0.13 | 6.55E-03 | 1.35 | 0.13 | 1.96E-02 | 1.13 | 0.14 | 3.67E-01 | 1.09 | 0.19 | 6.67E-01 | | 1.03 | 0.18 | 8.54E-01 | 1.24 | 0.07 | 1.36E-03 |
|  |  |  |  |  |  |  |  |  |  |  |  |  |  |  |  | |  |  |  |  |  |  |
|  |  |  |  |  | **355/437** | |  | **232/773** | |  | **159/831** | |  | **300/49** | | |  | **222 trios** | |  |  |  |
| **D** | rs17084051 | 54782338 | A | 1.42 | 0.13 | 7.99E-03 | 1.31 | 0.12 | 2.57E-02 | 1.12 | 0.14 | 4.13E-01 | 1.33 | 0.28 | 3.15E-01 | | 1.06 | 0.17 | 7.32E-01 | 1.25 | 0.07 | 8.06E-04 |
|  | rs7677751 | 54819217 | T | 1.56 | 0.13 | 9.47E-04 | 1.32 | 0.12 | 2.57E-02 | 1.12 | 0.14 | 4.48E-01 | 1.33 | 0.28 | 3.17E-01 | | 1.13 | 0.17 | 4.86E-01 | 1.29 | 0.07 | 1.50E-04 |
|  | rs2307049 | 54824911 | A | 1.48 | 0.14 | 4.37E-03 | 1.31 | 0.12 | 2.60E-02 | 1.12 | 0.14 | 4.35E-01 | 1.09 | 0.27 | 7.42E-01 | | 1.14 | 0.18 | 4.69E-01 | 1.26 | 0.07 | 6.85E-04 |
|  | rs7660560 | 54829151 | A | 1.49 | 0.14 | 3.94E-03 | 1.32 | 0.12 | 2.45E-02 | 1.10 | 0.14 | 5.20E-01 | 1.10 | 0.27 | 7.28E-01 | | 1.16 | 0.18 | 4.17E-01 | 1.26 | 0.07 | 6.87E-04 |
|  | rs2228230 | 54846797 | T | 1.45 | 0.16 | 1.87E-02 | 1.40 | 0.14 | 1.75E-02 | 1.12 | 0.14 | 4.33E-01 | 1.45 | 0.35 | 2.89E-01 | | 1.00 | 0.19 | 1.00E+00 | 1.26 | 0.07 | 2.40E-03 |
|  | rs4864872 | 54847041 | T | 1.45 | 0.16 | 1.87E-02 | 1.40 | 0.14 | 1.75E-02 | 1.12 | 0.14 | 4.33E-01 | 1.45 | 0.35 | 2.89E-01 | | 1.00 | 0.19 | 1.00E+00 | 1.26 | 0.07 | 2.40E-03 |
|  | rs3690 | 54856570 | C | 1.45 | 0.16 | 1.88E-02 | 1.42 | 0.14 | 1.27E-02 | 1.12 | 0.14 | 4.42E-01 | 1.44 | 0.35 | 2.93E-01 | | 1.03 | 0.18 | 8.54E-01 | 1.27 | 0.07 | 1.60E-03 |
