# Supplementary material for: Genome-Wide Meta-Analysis of Five Asian Cohorts Identifies PDGFRA as a Susceptibility Locus for Corneal Astigmatism
Source: PLoS Genet. 2011 Dec 1;7(12):e1002402. doi: 10.1371/journal.pgen.1002402 (PMC3228826; doi:10.1371/journal.pgen.1002402)
Supplement: Table S4 — Top SNPs (P-value≤1×10^−5^) identified from the meta-analysis of five GWAS with corneal astigmatism as a quantitative trait. A*: effect allele; no allelic effect size (β) estimated for STARS (parent-trios design) using FBAT on family-based quantitative trait. (DOCX) [file pgen.1002402.s014.docx]

**Table S4**

|  |  |  | |  |  | |  | **SP2** |  |  | **SiMES** | | |  | |  | **SINDI** | |  | **SCORM** |  |  | | **STARS*** | | | **Meta-analysis** |  |
| --- | --- | --- | --- | --- | --- | --- | --- | --- | --- | --- | --- | --- | --- | --- | --- | --- | --- | --- | --- | --- | --- | --- | --- | --- | --- | --- | --- | --- |
| **SNP** | **GENE** | **CHR** | | **BP** | **A** | | **β** | **s.e.** | ***P*** | **β** | **s.e.** | | | ***P*** | | **β** | **s.e.** | | ***P*** | **β** | **s.e.** | ***P*** | | **Z** | | ***P*** | ***P*** | |
| rs7677751 | PDGFRA | 4 | 54819217 | | T | -0.12 | | 0.04 | 1.33E-03 | -0.11 | | 0.03 | 2.58E-03 | | -0.09 | | 0.04 | 9.78E-03 | | -0.06 | 0.06 | 2.97E-01 | -1.09 | | 2.78E-01 | | 1.76E-07 | |
| rs7660560 | PDGFRA | 4 | 54829151 | | A | -0.12 | | 0.04 | 3.72E-03 | -0.10 | | 0.03 | 2.66E-03 | | -0.10 | | 0.03 | 5.96E-03 | | -0.05 | 0.06 | 4.21E-01 | -1.05 | | 2.94E-01 | | 3.41E-07 | |
| rs2307049 | PDGFRA | 4 | 54824911 | | A | -0.11 | | 0.04 | 4.94E-03 | -0.10 | | 0.03 | 3.19E-03 | | -0.10 | | 0.03 | 4.37E-03 | | -0.05 | 0.06 | 3.85E-01 | -1.02 | | 3.09E-01 | | 4.41E-07 | |
| rs17084051 | | 4 | 54782338 | | A | -0.11 | | 0.04 | 4.53E-03 | -0.11 | | 0.03 | 2.30E-03 | | -0.08 | | 0.03 | 2.61E-02 | | -0.07 | 0.05 | 2.30E-01 | -0.72 | | 4.74E-01 | | 2.15E-06 | |
| rs3802572 | CAMK1D | 10 | 12845383 | | C | 0.09 | | 0.03 | 7.16E-03 | 0.09 | | 0.03 | 4.92E-03 | | 0.07 | | 0.03 | 2.54E-02 | | -0.02 | 0.05 | 7.04E-01 | 0.90 | | 3.67E-01 | | 5.19E-06 | |
| rs2027924 |  | 8 | 18028003 | | A | -0.03 | | 0.07 | 6.54E-01 | 0.11 | | 0.05 | 1.84E-02 | | 0.15 | | 0.04 | 7.76E-05 | | 0.21 | 0.11 | 4.74E-02 | 0.77 | | 4.41E-01 | | 8.70E-06 | |

A: effect allele; No allelic effect size (β) estimated for STARS (parent-trios design) using FBAT on family-based quantitative trait.
