# Supplementary material for: Genome-Wide Meta-Analysis of Five Asian Cohorts Identifies PDGFRA as a Susceptibility Locus for Corneal Astigmatism
Source: PLoS Genet. 2011 Dec 1;7(12):e1002402. doi: 10.1371/journal.pgen.1002402 (PMC3228826; doi:10.1371/journal.pgen.1002402)
Supplement: Text S1 — Detailed QC procedures for SP2, SiMES, SINDI, SCORM and STARS. (DOCX) [file pgen.1002402.s015.docx]

Supplementary Text

SP2: Of the 2,867 blood-derived DNA samples, 392 samples were genotyped on the HumanHap 550v3, 1,459 samples on the 610-Quad, 817 samples on the 1M-Duov3, 191 samples on both 550v3 and 1M-Duov3, and 8 samples on both 610-Quad and 1M-Duov3. For the samples that were genotyped on two platforms, we used the genotypes from the denser platform in our study. We excluded 443 individuals on the following conditions, sample call rates of less than 95%, excessive heterozygosity, cryptic relatedness by IBS, population structure ascertainment, and gender discrepancies as listed in the main text. This left 2,434 post-QC SP2 samples. During the SNPs QC procedure, we excluded SNPs with low genotyping call rates (> 5% missingness) or monomorphic, with MAF < 1%, or with significant deviation from HWE (P< 10^-6^). This yielded a post-QC set of 462,580 SNPs. As SP2 samples are genotyped on different platforms, the concordance of the duplicate samples plated on different Beadarrays chips was also examined as quality of genotyping. The average SNP concordance rate between chips for the post-QC duplicated samples was 0.995. We additionally assessed the SNPs that are present on different platforms for extreme variations in allele frequencies with a 2-degree of freedom chi-square test of proportions, removing 62 SNPs with *P*-values < 0.0001. We further excluded those with missing phenotype data on corneal astigmatism (n=418). This yields a final set of 462,518 SNPs that are common to all 2,016 SP2 samples (1231 cases, 785 controls).

SiMES: Using the same quality control criteria, we omitted a total of 530 individuals from the total of 3,280 samples in the study, including those of subpopulation structure (n=170), cryptic relatedness (n=279), excessive heterozygosity or high missingness rate > 5% (n=37), and gender discrepancy (n=44). After the removal of the samples, SNP QC was then applied on a total of 579,999 autosomal SNPs for the 2,542 post-QC samples. SNPs were excluded based on (i) high rates of missingness (> 5%) (n=26,343); (ii) monomorphic SNPs or MAF < 1% (n=34,891); or (iii) genotype frequencies deviated from HWE (p <1 × 10^-6^) (n= 3,053). This yielded 515,712 SNPs after the same SNP QC criteria. Those with missing data or having cataract surgery were further removed (n=304). Finally, 515,712 SNPs in 2,238 samples (1018 cases, 1220 controls) were available for association analysis.

SINDI: We excluded 415 subjects from the total of 2,953 genotyped samples based on: excessive heterozygosity or high missingness rate > 5% (n=34) , cryptic relatedness (n=326), issues with population structure ascertainment (n=39) and gender discrepancies (n=16). This left a total of 2,538 individuals with 579,999 autosomal SNPs. During SNP QC procedure, i) 17,923 SNPs were removed due to high rates of missingness (> 5%), ii) and 17,966 SNP that were monomorphic or had MAF < 1%, iii) and 2,958 SNPs out of HWE (p < 10^-6^). After SNP QC, 541,152 SNPs were available for analysis. We further excluded any individuals with missing data on corneal astigmatism measurements and having history of cataract surgery (n=399). This yielded a final dataset of 2,139 individuals (825 cases, 1,314 controls) and 541,152 post-QC SNPs.

SCORM: A total of 1116 DNA samples (1037 from buccal swab and 79 from saliva) were genotyped on the Illumina HumanHap 550 Beadchips or 550 Duo Beadarrays. 187 samples were excluded including i) 70 for low call rates below 98%, ii) 6 for poor genotyping quality, iii) 11 for sib-ships, vi) 18 with inconsistent gender information, v) 3 for population structure, and vi) 79 without AL measurements and demographic data. This left a total of 929 samples for further SNP QC. Based on 514,849 autosomal SNPs, we excluded 31,457 markers if they had missing genotype calls > 10%, a minor allele frequency < 1%, or significantly deviated from HWE (p < 10^-6^). In summary, a final set of 929 samples (760 cases, 169 controls) with 483,392 post-QC SNPs were included in the analysis.

STARS: Of the total of 1,451 genotyping samples, the individuals were excluded based on gender discrepancy (n=17), high missingness (> 5%), excessive heterozygosity (n=11) and excessive Mendelian inconsistency (Mendelian error per family > 1%; n=14). This left a set of 1,408 samples in 436 families after quality control, of which 57 samples (from 29 families) were further excluded due to founder singleton, or non-founder without 2 parents, yielding 1,351 individuals in 407 families. SNP QC was performed on the 576,979 autosomal SNPs by excluding those with high missingness rate > 5% (n=1,984), gross departure of HWE for parents (P < 10^-8^) (n=908), monomorphic SNPs and SNPs with MAF <1% (n=80,804), and SNPs exhibiting significant degree of Mendelian inconsistencies (> 10% of all the trios) (n=7). This led to a final set of 493,594 post-QC SNPs. We further restricted the family-based analyses to 1,191 individuals in 397 parents-trios (317 nuclear families) having corneal astigmatic child. In all, 493,594 SNPs for 1,191 samples from 397 parent-trios were available for family GWAS on corneal astigmatism.
